# A model of temporal scaling correctly predicts that motor timing improves with speed

**DOI:** 10.1038/s41467-018-07161-6

**Published:** 2018-11-09

**Authors:** Nicholas F. Hardy, Vishwa Goudar, Juan L. Romero-Sosa, Dean V. Buonomano

**Affiliations:** 1 Neuroscience Interdepartmental Program, University of California Los Angeles, Los Angeles, CA 90095 USA; 2 Departments of Neurobiology, University of California Los Angeles, Los Angeles, CA 90095 USA; 3 Departments of Psychology, University of California Los Angeles, Los Angeles, CA 90095 USA

## Abstract

Timing is fundamental to complex motor behaviors: from tying a knot to playing the piano. A general feature of motor timing is temporal scaling: the ability to produce motor patterns at different speeds. One theory of temporal processing proposes that the brain encodes time in dynamic patterns of neural activity (population clocks), here we first examine whether recurrent neural network (RNN) models can account for temporal scaling. Appropriately trained RNNs exhibit temporal scaling over a range similar to that of humans and capture a signature of motor timing, Weber’s law, but predict that temporal precision improves at faster speeds. Human psychophysics experiments confirm this prediction: the variability of responses in absolute time are lower at faster speeds. These results establish that RNNs can account for temporal scaling and suggest a novel psychophysical principle: the Weber-Speed effect.

## Abstract (web summary)

Humans can perform complex motor movements at varying speeds. Here, the authors show that a recurrent neural network can be trained to exhibit temporal scaling obeying Weber’s law as well as validate a prediction of the model of improved precision of movements at faster speeds.

## Introduction

It is increasingly clear that the brain uses different mechanisms and circuits to tell time across different tasks. For example, distinct brain areas are implicated in sensory^1,2^ and motor^3–6^ timing tasks on the scale of hundreds of milliseconds to a few seconds. This multiple clock strategy likely evolved because different tasks have distinct computational requirements. For example, judging the duration of a red traffic light requires estimating absolute durations, but tying your shoe and playing the piano rely on the relative timing and order of activation of similar sets of muscles. A general property of these complex forms of motor control is temporal scaling: well-trained motor behaviors can be executed at different speeds. Despite the importance of temporal scaling in the motor domain, basic psychophysical and computational questions remain unaddressed. For example, is temporal scaling intrinsic to motor timing? In other words, once a complex pattern is learned can it be accurately sped-up or down, like changing a movie’s playback speed?

The neural mechanisms underlying temporal scaling remain unknown in part because motor timing itself is not fully understood. Converging evidence from theoretical^7–9^ and experimental studies suggests that motor timing is encoded in patterns of neural activity, i.e., population clocks^4,5,10–15^. Although numerous computational models have been proposed to account for timing^16,17^, temporal scaling remains largely unaddressed.

Here, we show that RNNs can be trained to exhibit temporal scaling. The model also accounts for a signature of motor timing known as the scalar property (Weber’s law): the standard deviation of timed responses increases linearly with time^18^. However, the model predicts that the relationship between variance and time is not constant, but dependent on speed. A psychophysical study in which humans produce a complex pattern of taps confirms this prediction: precision is better at the same absolute time when a motor pattern is being produced at a higher speeds.

## Results

### Temporal scaling of complex motor patterns

**Fig. 1:**
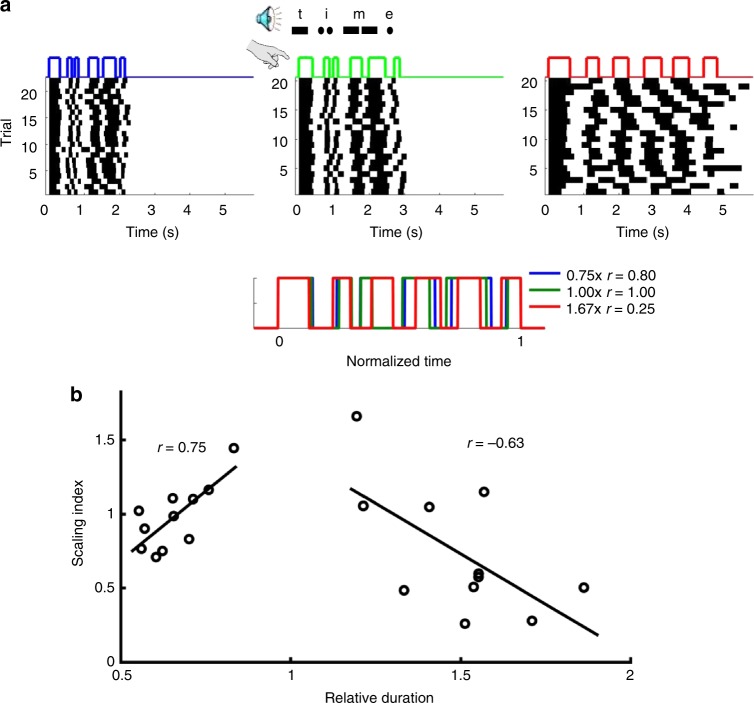
Limited temporal scaling of a learned Morse code pattern. Subjects were trained to tap the Morse code for “time” at a speed of 1× (10 wpm) over four consecutive days (Methods). **a** On the fifth day, subjects were asked to produce the pattern at three different speeds: twice as fast (2×), normal speed (1×), and twice as slow (0.5×) (data from a single subject). Bottom: Average of the responses above plotted in normalized time. The legend indicates the produced speed relative to the trained (1×) condition and the correlation of the mean response to the response at trained speed. **b** The relationship between produced speed and temporal scaling accuracy for all 11 subjects. There was a significant correlation between speed and accuracy for both the fast (*r* = 0.75, *p* = 0.008, two-tailed *t*-test) and slow (*r* = −0.63, *p* = 0.038, two-tailed *t*-test) patterns

Humans can execute well-trained complex movements such as speaking or playing a musical instrument at different speeds. However, it is not clear how well complex temporal patterns can be automatically executed at different speeds. A few studies have examined temporal scaling in humans^19,20^, however, to the best of our knowledge no studies have trained subjects to learn aperiodic temporal patterns at a single speed, across days, and examined the subject’s ability to reproduce that pattern at faster and slower speeds. We thus first addressed whether temporal scaling is an intrinsic property of motor timing by training subjects on a temporal pattern reproduction task (Methods). To ensure that any temporal scaling was not the result of previous experience, subjects learned to tap out a Morse Code pattern (the word “time”) at a speed of 10 words-per-minute (the duration of a “dot” was 120 ms). The target pattern was composed of six taps and lasted 2.76 s (Fig. 1a).

After training for 4 days, subjects were asked to produce the pattern at the original speed, twice as fast (50% duration), and at half speed (200% duration) under freeform conditions—i.e., they were not cued with any target pattern during this test phase. At the 1× speed subjects produced the target pattern with a performance score (correlation between the produced and target patterns) of 0.66 ± 0.04. As expected in a freeform condition, there was significant variability in the produced speeds and few subjects reached the speeds of 2× and 0.5×. Thus we were able to measure how well subjects scaled the trained pattern, and the relationship between performance and speed. We quantified temporal scaling using a scaling index based on the time normalized correlation (Methods) between the 1× and scaled patterns (Fig. 1b). The scaling index and overall pattern duration for both the fast (short) and slow (long) patterns were highly correlated (*r* = 0.75, *p* = 0.008; and *r* = −0.63, *p* = 0.038, respectively). Furthermore, the normalized RMSE (NRMSE) tended to be smaller for the trained 1× speed, and most of the NRMSE was attributable to the standard deviation as opposed to the bias (i.e., the difference in the average response and target times; Supplementary Fig. 1). These results confirm that with moderate levels of training, humans are intrinsically able to speed up or slow down a learned motor pattern, but that performance progressively degraded at untrained speeds.

### RNN model of motor timing

How can neural circuits generate similar temporal patterns at different speeds? To examine the potential mechanisms of temporal scaling, we turned to a population clock model of timing that has previously been shown to robustly generate both simple and complex temporal patterns^8^. The model consisted of an RNN with randomly connected firing rate units whose initial weights were relativity strong, placing the network in a high-gain (chaotic) regime, in which networks exhibit complex (high-dimensional) activity. In theory, this activity can encode time while retaining long-term memory on scales much longer than the time constants of the units. In practice, however, this memory is limited by chaotic dynamics^21^. Chaotic behavior impairs networks’ computational capacity because the activity patterns are not reproducible in noisy conditions. It is possible, however, to tune the recurrent weights to tame the chaos while maintaining complexity (Methods). The result is the formation of locally stable trajectories, i.e., dynamic attractors, that robustly encode temporal motor patterns. We first asked whether these RNNs can account for temporal scaling.

**Fig. 2:**
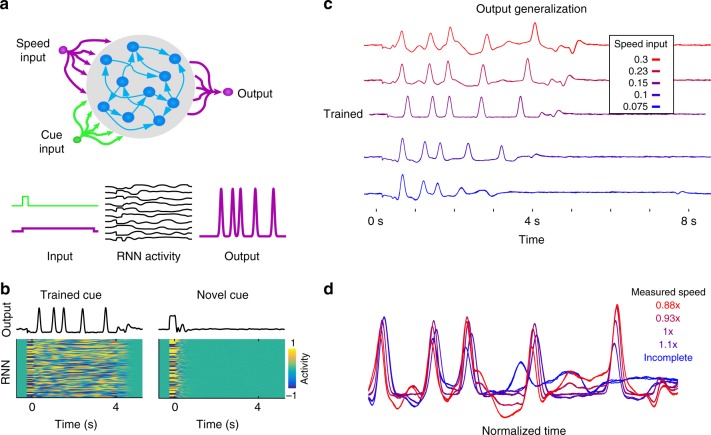
Robust temporal scaling is not produced by altered input drive of a RNN model. **a** The model was composed of recurrently connected firing rate units, which received two external inputs and connected to a single output. One input served as a start cue and was active briefly at the start of each trial between *t* = [−250, 0]ms. The second input delivered a constant low amplitude speed signal for the duration of a trial. **b** The RNN was trained to autonomously produce a neural trajectory lasting four seconds at 1× speed (*y*^SI^ = 0.15). At the end of the trajectory, the recurrent network was trained to return to a rest state (*r* = 0), forming a gated attractor: networks only generate long-lasting stable dynamic activity in response to the trained cue. Following recurrent training, the output unit was trained to produce a series of five taps at 325, 1025, 1500, 2400, and 3500 ms. In response to a novel cue input the RNN activity does not enter the trained dynamic attractor, and activity quickly returns to rest. **c** Networks trained at one speed do not scale the speed of their dynamics according to changing input drive. The speed signal was varied between *y*^SI^ = [0.3,0.23,0.15,0.1,0.075]. **d** Traces shown in **c** plotted in normalized time

An intuitive mechanism for temporal scaling is that increased external drive onto a network increases the speed of its dynamics. Thus, to test whether these RNNs could account for temporal scaling, we examined the effects input drive on speed. The RNNs received two independent inputs: one transient cue to start a trial and a second tonic speed input (*y*^SI^) to modulate the speed of the dynamics. The recurrent units generate motor patterns through synapses onto a single output unit (Fig. 2a).

We trained chaotic RNNs to reproduce, with significant injected noise, an innate pattern of network activity (i.e., one it produced before any weight modification) while receiving a fixed amplitude speed input (defined as speed 1×, *y*^SI^ = 0.15), then trained the output to produce an aperiodic pattern composed of five so-called taps after the cue offset (Methods). Unlike biological motor systems, RNNs in high-gain regimes are typically spontaneously active, i.e., their activity is self-perpetuating. To increase the model’s congruence with cortical dynamics and motor behavior, we developed a method of training the recurrent units to enter a rest state when not engaged in a cued task. In this procedure, the recurrent units are trained to maintain a firing rate of approximately zero after the target pattern has terminated (Methods). This training produces a gated dynamic attractor: in response to a cued input the network produces the trained dynamics and then returns to a rest state (Fig. 2b). In contrast, in response to an untrained input the network activity quickly decays to the rest state. Consistent with the lack of spontaneous activity the real eigenvalues of the trained weights are less than one (Supplementary Fig. 2).

**Fig. 3:**
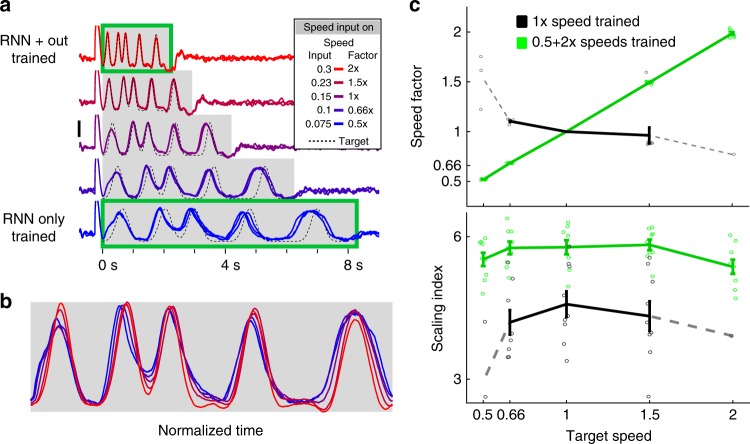
RNNs trained at multiple speeds exhibit robust temporal scaling. **a** Output activity of an RNN trained to produce the scaled patterns of recurrent activity at 0.5× (*y*^SI^ = 0.075) and 2× (*y*^SI^ = 0.3) speeds. The output was trained only at the 2× speed. After training (weight modifications stopped), the network was tested at different input speed levels (*y*^SI^ = [0.075,0.1,0.15,0.23,0.3])—corresponding to speeds of 0.5, 0.66, 1, 1.5, and 2×. Three example test trials at each speed are overlaid. **b** One trial from each test speed above shown with time normalized to the end of the active period. **c** Networks (*n* = 10) trained at two speeds generalize to untrained speed inputs. Top: The speed factor (the mean ratio of the final tap at each speed to the mean final tap time at 1× speed over 20 trials) of networks trained at two speeds (green), and one speed (black). Bottom: The scaling index of networks trained on two speeds is higher than those trained on one speed. Error bars represent SEM (*N* = 10), and circles show the value for each network. Because the activity of the one-speed networks degrades at more extreme speeds as shown in Fig. 1, many networks did not produce detectable taps (output peaks) at extreme speeds and we, therefore, could not calculate a scaling index or index for them. We show in dotted lines the values for the networks that completed at least one trial at the extreme speeds

After training, the network was able to reproduce the target output at the trained speed. However, when tested at a range of speeds—by changing the tonic speed input—the network exhibited limited temporal scaling (Fig. 2c). Notably, these scaled patterns degraded substantially (Figs. 2c, d and 3). This establishes that simply changing the amplitude of a tonic input cannot account for the factor of four range of temporal scaling observed in humans.

### RNN model of temporal scaling predicts a Weber-Speed effect

We next examined whether temporal scaling could be learned by training RNNs to produce the same pattern of activity in the recurrent units at two different speeds (0.5× and 2×, Methods). After the recurrent network was trained, we trained the output to produce the same pattern as in Fig. 2, but only at the 2× speed. Given the highly nonlinear—and initially chaotic—nature of these RNNs it was unclear if they would scale to novel speed inputs. But the results show that when tested at different speed levels, networks exhibited robust linear scaling (Fig. 3a, b). Note that because the output was trained only at 2× speed, any change in the speed of the output reflects an underlying change in the speed of recurrent activity. Compared to RNNs trained on a single speed, those trained on two speeds accurately interpolated their activity between the trained speeds. As mentioned there was a small degree of “intrinsic” temporal scaling in the RNNs trained at one speed (black lines in Fig. 3c), however, the scaling was very limited (0.9× to 1.15×, a factor of approximately 1.25). In contrast, when trained on two speeds RNNs accurately interpolated over a factor of 4, and even at speeds outside the trained range, there was some temporal scaling (Supplementary Fig. 3).

**Fig. 4:**
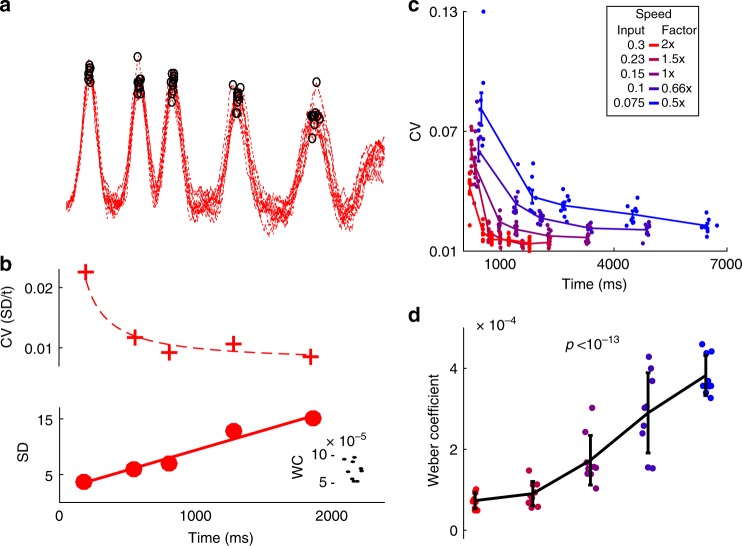
RNN models of temporal scaling predict a novel Weber-Speed effect. **a** Ten trials of the output activity of one network at 0.5× speed with tap times indicated by black circles. **b** Trained RNNs account for generalized Weber’s Law, which predicts a linear relationship between the mean and standard deviation of timed intervals. Top: The coefficient of variation (CV, SD/*t*) at each of the five taps shown in **a**. The dotted line shows the CV calculated using the fit below. Bottom: standard deviation linearly increases with time. Line shows the linear fit (*r*^2^ = 0.96). Inset shows the Weber Coefficient (the slope of variance vs. mean time) at 0.5× speed for all ten trained networks. **c** The CV of ten networks calculated from 20 trials at each tested speed. Note that at the same absolute time across speeds, the CV is higher when speed is slower (the Weber-Speed effect). **d** The Weber Coefficient increases at slower speeds (Repeated-measures one-way analysis of variance; *F* = 54.4, *p* < 10^−13^). Networks (*n* = 10) for this analysis were trained and tested at 0.25 noise amplitude. Error bars represent SEM

Because Weber’s law is often held as a benchmark for timing models^17^, we examined whether the SD of the model’s across-trial tap times was linearly related to absolute (mean) time. There was a strong linear relationship between SD and time, (Fig. 4b), allowing us to calculate the Weber coefficient (slope of the variance vs. *t*^2^). In contrast to other timing models—drift-diffusion models for example^22^—RNNs inherently account for Weber’s law. This is in part because the recurrent nature of these networks can amplify noise, imposing long-lasting temporal noise correlations, leading to near linear relationships between SD and time^23^ (Supplementary Fig. 4)

Speed was negatively correlated with both coefficient of variation (CV or Weber fraction, Fig. 4c), and Weber coefficient (Fig. 4d). Specifically, the lower the speed the higher the Weber coefficient. Moreover, this effect was robust to changes in network size, noise amplitude, and whether networks were trained to speed-up or slow-down at higher input amplitudes (Supplementary Fig. 5). This counterintuitive observation implies that at the same absolute time temporal precision is significantly lower at slower speeds. To use an analogy: a clock would be more precise at timing a two second interval when that interval was part of a short (high speed) pattern compared to a two second interval that was part of a long (slow) pattern. In other words, the model predicts that humans are less precise halfway through a four second pattern than at the end of the same pattern produced twice as fast. We will refer to this speed-dependent improvement in temporal precision as the Weber-Speed effect (and address its potential relationship with the subdivision effect below).

The learning rule used to train the RNNs provides a robust means to generate complex and highly stable spatiotemporal patterns (but is not meant to represent biologically realistic learning rule in recurrent neural networks). It is possible that the Weber-Speed effect observed above emerged from some property specific to the training. Thus, we also examined timing in RNNs trained with Hessian-free backpropagation through time (BPTT)^24,25^ and a standard echo-state network^26,27^. Compared to innate learning, these training algorithms were not as well-suited to learning the same complex long-lasting aperiodic temporal patterns across speeds. Indeed, training RNNs to produce the aperiodic output with BPTT did not result in robust generalization (at least under the parameter conditions used here), but networks using a simple linear ramping output generalized their speed via parallel neural trajectories (Supplementary Fig. 6). Importantly, both rules generated RNNs that exhibited a Weber-Speed effect at the trained speeds (Supplementary Fig. 6). Thus, our results suggest that the Weber-Speed effect is a robust property of timing generated by the dynamics of RNNs (Supplementary Fig. 7).

### Humans exhibit the Weber-Speed effect

**Fig. 5:**
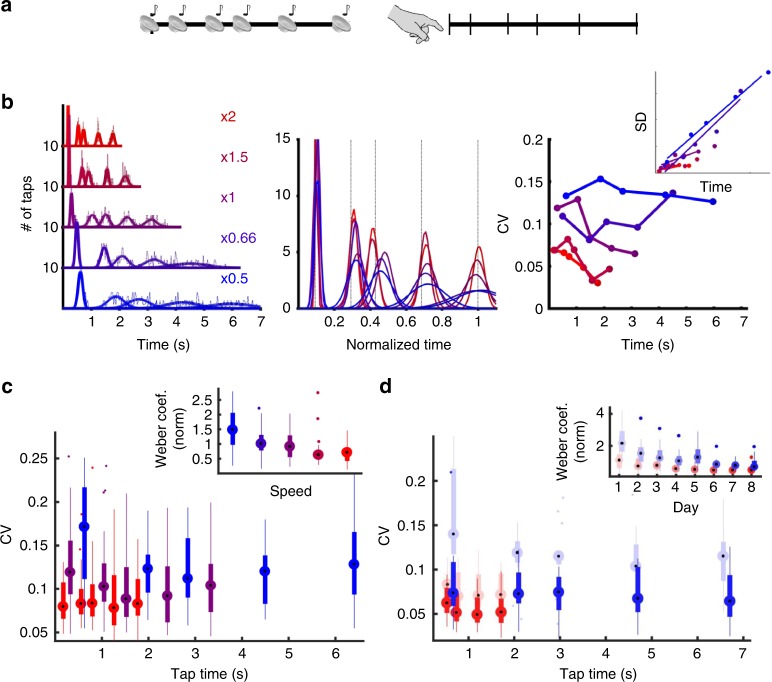
Test of the Weber-Speed effect prediction. **a** Subjects were trained on an auditory temporal pattern reproduction task, using the same aperiodic pattern and same five speeds used to test the RNNs. **b** Left: Histogram (dashed lines) and Gaussian fits (solid lines) of the cued taps at all five speeds from a single subject (bin sizes scale with target duration). Middle: the fits shown with time normalized to the mean of the last tap (vertical lines represent target times)—note that the scaled fits do not overlap as expected by Weber’s law. Right: CV of each tap at each speed, with SD vs. mean time inset. The slope of the linear fit of the variance vs. *t*^2^ corresponds to the Weber coefficient (SD vs. time is shown for visualization purposes). **c** Whisker plots of the CV of all subjects (*n* = 25) for three of the five speeds (0.5×, 1×, and 2×). Note that, as in the RNN model, the CV at the same absolute time is higher at slower speeds. Inset shows the Weber coefficient for all five speeds. **d** The Weber-Speed effect is not due to inexperience with the task. A subset of 14 subjects were trained to produce the 0.5× and 2× slow speeds over eight additional days. The Weber-Speed effect persists over the course of training. CVs are shown for the first (light) and last (dark) day of training for both speeds. Inset: the Weber coefficients across all 8 days of training. Whisker plots show the median, lower and upper quartile, 1.5× interquartile range, and outliers. The color scheme is that same as in Fig. 4

To the best of our knowledge, the notion that temporal precision is worse for complex temporal patterns produced at low speeds has never been predicted or experimentally tested. Thus, we tested this prediction using a temporal reproduction task in which subjects were required to reproduce an aperiodic pattern composed of six taps at five different speeds (the same pattern and speeds used model above). Subjects (*n* = 25) listened to an auditory pattern composed of six tones and were asked to reproduce it using a keypad (Fig. 5a, Methods). In each block subjects heard the pattern at one of five temporally scaled speeds (0.5×, 0.66×, 1×, 1.5×, and 2×) and reproduced the pattern (Fig. 5b, single subject). Based on the mean and SD of the taps it is possible to calculate the CV for each tap, and the Weber coefficient (inset Fig. 5b right, SD vs. *t* is shown for visualization). Across subjects (Fig. 5c) CVs were significantly different across speeds (*F*_4,96_ = 10.4, *p* < 10^-6^, speed effect of a two-way repeated ANOVA), and the Weber coefficient decreased with higher speed (*F*_4,96_ = 7.3, *p* < 0.001, one-way repeated ANOVA).

The above data is potentially confounded with task difficulty or learning—i.e., the difference in the Weber coefficients across speeds could potentially reflect some nonspecific effect in which slower patterns are harder to learn. We thus trained a subset of subjects (*n* = 14) on the fastest and slowest speeds over an 8-day period. Again, at the same absolute time the CV was lower for the faster speed across training days (e.g., ≈0.7 s in Fig. 5d). The Weber coefficient was significantly smaller for the faster speeds across training days (Fig. 5d, inset; *F*_1,13_ = 16.58, *p* < 0.002, speed effect two-way repeated ANOVA; pairwise posthoc test on each day, maximum *p* = 0.056, Tukey-Kramer)—even as subjects showed asymptotic learning, seen in the progressive decrease in the Weber coefficients across days. To further confirm the Weber-Speed effect and examine its dependence on training we performed a second study in which subjects (*n* = 14) were trained on three speeds (0.5×, 1×, and 2×) across 3 days. Analysis of the Weber coefficient across speeds and days again revealed a robust effect of speed (Supplementary Fig. 8, *F*_2,28_ = 11, *p* < 0.0005, two-way repeated ANOVA) as well as an improvement across days (*F*_2,28_ = 7.1, *p* < 0.005), and no significant interaction between speed and day of training. These results confirm that temporal precision is better at faster speeds.

### Speed or subdivision?

**Fig. 6:**
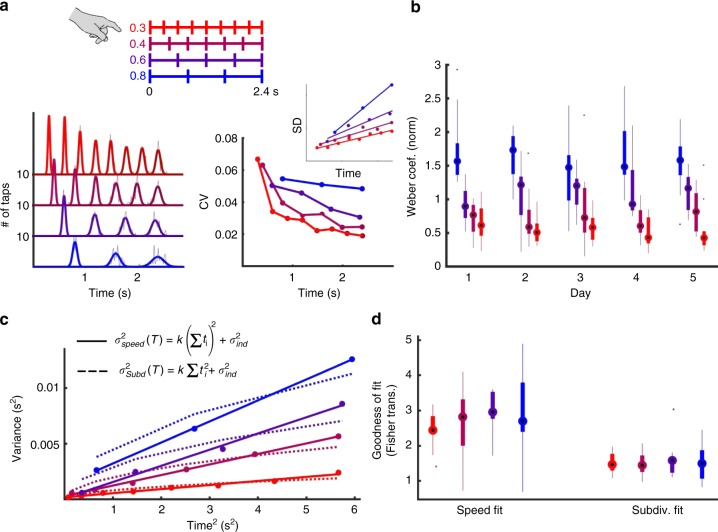
Comparison of the Weber-Speed and subdivision hypotheses using a periodic task. Subjects were trained on four periodic auditory temporal patterns all lasting 2.4 s (periods of 0.3, 0.4, 0.6, and 0.8 s) over 5 days. **a** Left: Histogram (dashed lines) and Gaussian fits (solid lines) of the taps at all four speeds for a single subject. Right: CV of each tap at each speed, with SD vs. mean time shown as the inset. **b** Whisker plots of the Weber coefficient of all subjects (*n* = 11) across the 5 days of training. **c** Example fits of the variance at time *T* composed of *n* subintervals (*t*_1_, *t*_2, …,_ *t*_*n*_) according to the speed (continuous, solid lines) and subdivision (reset, dashed lines) hypotheses ($\sigma _{\mathrm{ind}}^2$ represents the time independent source of variance). **d** Goodness of fit values (Fisher-transformed *r*^2^) for both the speed and subdivision hypotheses for each speed across all subjects (data shown are from the last day of training)

The Weber-Speed effect is potentially related to the so-called subdivision effect. Specifically, it is well-established that the timing of a given absolute interval can be improved by subdividing that interval into smaller subintervals—e.g., by tapping your foot or counting—can improve the timing of a longer interval^28,29^. Subdivision cannot account for the Weber-Speed effect in the model because the internal dynamics is independent of what the output unit is trained to do, but it could explain the psychophysical results because the subintervals of the pattern are shorter at higher speeds. To directly compare both the speed and subdivision hypothesis in the psychophysics experiments we trained subjects on a periodic subdivision task over 5 days. Subjects produced a series of taps with a total duration of 2400 ms, with four different inter-tap intervals (speeds; Fig. 6a). Similar to results from the aperiodic temporal pattern, subjects showed reduced variability at the same absolute time when the inter-tap-interval was shorter (Fig. 6b). Here, the subdivision and speed hypotheses are confounded, but can be dissociated based on the standard explanation of the subdivision effect. Subdivision is hypothesized to improve timing because a central clock is reset at each tap^30^, whereas in our population clock model timing of a complex pattern relies on a continuous timer. In the case of a single interval both views generate the same variance, but in the case of a pattern composed of a sequence of intervals (*t*_1_, *t*_2_, …, *t*_*n*_) they generate different variance signatures (Fig. 6c). Specifically, the standard interpretation of the subdivision (reset) effect is that the total variance is a function of the sum of the component intervals squared, whereas under the speed (continuous) perspective the variance is a function of the absolute time squared. In other words, the Weber-Speed interpretation predicts that the SD vs. time relationship should be linear for all taps at a given speed, while subdivision predicts a sublinear relationship. We fit each subject’s responses assuming either a speed or subdivision interpretation of Weber’s generalized law. While both fits captured the data well, the goodness-of-fit of the speed prediction was significantly better (Fig. 6c, d, fits for day 5 shown, *F*_1,10_ = 48, *p* < 10^−4^, two-way repeated ANOVA on Fisher-transformed *r*^2^ values). A similar analysis for the results of the aperiodic psychophysical experiment presented in Fig. 5 also revealed that the speed fit was significantly better than the reset fit (Supplementary Fig. 9). These results suggest that the standard subdivision effect may be best interpreted not as the result of resetting an internal timer but rather of increasing the speed of the internal dynamics of a population clock.

### Mechanisms of temporal scaling

Having established and tested a model of temporal scaling, we next used the model to examine potential network-level mechanisms underlying temporal scaling. At first glance the notion that an RNN can generate the same trajectory at different speeds is surprising, because it seems to imply that different tonic inputs can guide activity through the same points in neural phase space at different speeds. Furthermore, it is important to emphasize that the relationship between input amplitude and speed is arbitrary: the model exhibits temporal scaling whether the network is trained so that larger speed inputs increase or decrease trajectory speed (Supplementary Fig. 5g), implying that temporal scaling is an emergent phenomenon. Additionally, untrained RNNs will not scale when the tonic input changes. In the chaotic regime used here, any change in input produces dramatically different trajectories, and even when trained on one speed the network did not exhibit robust scaling (Figs. 2, 3). Furthermore, when RNNs were trained on two speeds with BPTT robust temporal scaling was not observed (Supplementary Fig. 6).

**Fig. 7:**
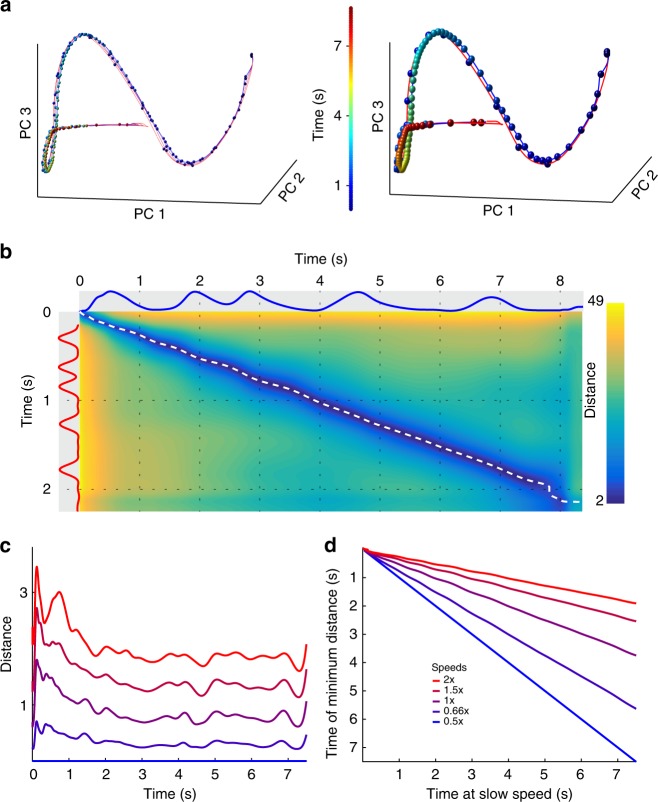
Temporal scaling relies on parallel neural trajectories at different speeds. **a** Trajectory of RNN activity at five speeds projected onto the first three principal components. Right: same data, but only the slowest (blue line) and fastest (red) speeds are plotted to highlight the difference in speed of the two trajectories. Colored spheres indicate absolute time in each trajectory (100 ms between spheres), and reveal the differences in the speeds of the trajectories in neural phase space. **b** Euclidean distance matrix between the fast and slow trajectories in neural space at each point in time along each trajectory (network size: *N* = 1800). Blue and red traces along the axes show the output. White dotted line traces the minimum distance between the two trajectories, which never reaches zero. **c** The minimum distance along the slowest trajectory from each other speed. **d** The relative timing at which the minimum occurs in each trajectory. For example, at 4 s in the slowest speed (*x*-axis) the trajectory is closest to the 2× speed at 1 s

Because the network is trained to reproduce the same trajectory at two different speeds, the most straightforward way scaling to novel speeds could emerge is via parallel neural trajectories. But such a mechanism could take two forms: nearby trajectories that are traversed at different speeds, or distant trajectories that are traversed at the same speed. As a first step toward examining the underlying mechanisms we first visualized the trajectories in principal components analysis (PCA) space. This revealed that trajectories at different speeds follow offset paths of similar length through neural phase space that are traversed over different durations (Fig. 7a). In other words, the trajectories are arranged according to speed in an apparently parallel manner. To quantify this observation, we calculated the Euclidean distance in neural space (*n* = 1800) between the trajectory at each speed and the 0.5× speed (Fig. 7b). Finding the minimum distance between the comparison speed and the 0.5× speed revealed that the trajectories maintained a fairly constant distance from each other (Fig. 7c). Examining the times that the trajectories were closest also provided an unbiased estimate of the relative speed. For example, if the test trajectory is moving four times faster as the reference, they should be closest when the fast trajectory has been active for ¼ the elapsed time. In other words, plotting $t_{\mathrm{min}}^{2x}$ vs. $t_{\mathrm{elapsed}}^{0.5x}$ should form a line with slope 0.25, which is indeed what we observed. Moreover, this relationship generalized to novel interpolated speeds (Fig. 7d).

**Fig. 8:**
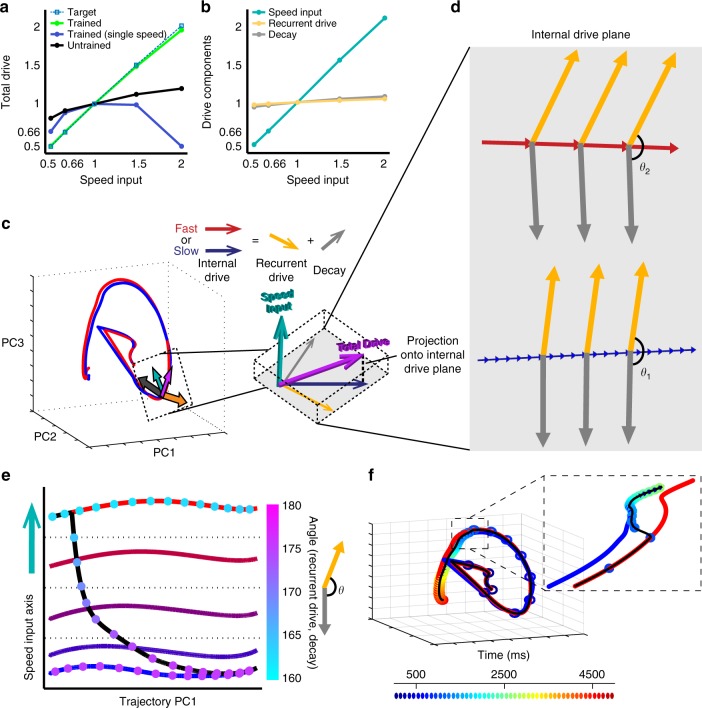
Mechanisms of temporal scaling in the RNN. **a** Magnitude of the instantaneous change in activity (trajectory speed) of the recurrent network (total drive) scales linearly with speed input value in networks trained at two speeds (green), but not in networks trained at one speed or untrained networks. Total drive is normalized to the 1× speed. **b** Decomposing network drive into its three components (recurrent, decay, and input) revealed that the recurrent and decay components do not individually scale with speed input, thus neither of them in isolation can account for temporal scaling. **c** To examine the relationship between the recurrent and decay components separate from the input drive, we projected them onto the internal drive plane, a subspace orthogonal to the speed input (Methods). **d** This projection revealed that at faster speeds the angle between the recurrent and decay components decreases, creating a second-order effect that drives the network activity along the trajectory more quickly. **e** Network activity projected onto the input axis and the first principal component of network activity (the dimension which accounts for the largest amount of variance). The colored markers indicate the angle between the recurrent and decay components. The position along the input axis does not change as a function of time, indicating that speed is encoded by the position along the input axis. When the speed input level is abruptly decreased partway through the trajectory (black line), the network switches from fast to slow speeds via an increase in the angle between the recurrent and decay components. **f** Neural trajectories in the first three principal components during a mid-trajectory change in speeds. As the dynamics transition from fast to slow (inset), the trajectory (black line) moves along a hyperplane defined by the parallel trajectories shown in Fig. 6

Given that the network was trained to reproduce the same trajectory at two speeds, it is not surprising that it converges to a solution with two nearby parallel trajectories. More interesting is that it is able to generalize to novel speeds, and how this is achieved. That is, how does changing the magnitude of a static input result in trajectory speeds that scale approximately linearly with the input magnitude? Understanding the underlying dynamics of complex nonlinear neural networks is a notoriously challenging problem with few tools available^25^. Here, we introduce a method to dissect the internal forces driving a network. We first quantified the total drive to the network: the time-dependent change in the total synaptic input onto each neuron in the RNN. Measuring the magnitude (Euclidean norm) of the total drive showed that—in contrast to untrained networks or to networks trained at a single speed—the total drive scaled with the cued speed (Fig. 8a). To address how the total drive scales the neural dynamics, we used a novel network drive decomposition method^31^. This approach decomposes the total network drive into its three components: recurrent synaptic drive, synaptic decay (which drives the network towards the origin), and the external tonic (time independent) speed input (Fig. 8c). While the speed input magnitude scaled with speed as defined by the experimental conditions, the recurrent and decay drive magnitudes did not, meaning that the recurrent and decay components in isolation cannot account for temporal scaling (Fig. 8b).

Analysis of the dynamics also revealed that, at each speed, the trajectories traversed directions that are independent of the speed input—i.e., the projection of each trajectory onto the speed input axis has low variance (explained variance was <1% at all speeds). There are two consequences to the observations that the time-varying dynamics are not driven by the input, and that the recurrent drive and decay magnitudes did not exhibit temporal scaling: (1) at each speed, some combination of these internal drive components counterbalance the speed input; and (2) they collectively underlie temporal scaling of the trajectory. To isolate the contribution of these interactions we studied the internal drive components in the subspace orthogonal to the speed input axis (Fig. 8c). Measurements showed that even in this subspace, changes of recurrent drive and decay magnitudes did not explain temporal scaling of the total drive. Instead, the recurrent synaptic drive and decay opposed each other (the angle between them is obtuse) throughout the trajectory, and the extent of this opposition altered the trajectory’s speed (Fig. 8d). Specifically, the angle between the two components decreases as the speed input increases (*θ*_2_ < *θ*_1_), amplifying the net (or total) drive.

Projecting the trajectories onto the speed input axis revealed that speed is encoded in the trajectory’s position rather than its direction (Fig. 8e). Moreover, by traversing phase space along directions that are independent of the speed input, the trajectory’s position with respect to the speed input stayed relatively constant, and thus so did actual speed. To confirm this, we asked if—as with biological motor patterns—a network could switch speeds mid-trajectory. Indeed, by decreasing the speed input in the middle of a fast (2×) trajectory we observed a rapid transition to the slow trajectory (Fig. 8f). Network drive decomposition showed that a change in the speed input caused an imbalance between it and the internal drive, altering the position of the trajectory along the speed input axis. In turn, this increased the angle between the recurrent and decay drives, slowing the trajectory down. It also rebalanced the speed input and the internal drive components such that trajectory speed stopped changing when the balance between input and internal drive was restored (Fig. 8e). Altogether, these results demonstrate that temporal scaling is the outcome of speed input-dependent balance between the recurrent and decay drives.

## Discussion

It is increasingly clear that on the scale of hundreds of milliseconds to seconds the brain represents time as dynamically changing patterns of neural activity (i.e., population clocks)^4,5,12,14,32^. Timing on this scale exhibits: (1) the ability to execute the same motor pattern at different speeds, and (2) a linear increase in motor variability with time (Weber’s law). Here, we unify and extend these observations by building an RNN that not only performs temporal scaling and accounts for Weber’s law, but also predicts that Weber’s law is speed-dependent. We tested this prediction using human psychophysics experiments, confirming that in absolute time the temporal precision of motor responses is dependent on speed.

Few studies have quantified temporal scaling of complex aperiodic motor patterns in humans^19,20^. However, studies in the sensory and sensory-motor domain have clearly established that interval discrimination learning does not generalize new intervals^33–36^. In a manner of speaking, temporal scaling of motor patterns (e.g., Fig. 1) represent generalization to different intervals. However, the difference between interval and pattern timing is significant^16^. Simple intervals are defined by their absolute duration—i.e., the difference between a scaled interval and a different interval is ambiguous—whereas patterns can be defined by the relationship of the component subintervals. Thus, the apparent difference between generalization of learned intervals and patterns could be related to different underlying neural mechanisms.

While Weber’s law is well-established in humans^37–39^, it’s neural underpinnings are debated^22^. Early internal clock models consisted of an accumulator that integrated the pulses of a noisy oscillator. In their simplest form, however, these models did not account for Weber’s law because the SD of such a clock follows $\sqrt t$ rather than *t*. Thus, early internal clock models postulated that Weber’s law arises from a second clock-independent noise source, such as the memory of the interval being generated^18,28^. Other models^22,40,41^, including those based on the variance between multiple timers, can intrinsically account for Weber’s law, but the biological plausibility of such variance-based models is unclear. Our results suggest that population clocks based on recurrent dynamics can intrinsically account for Weber’s law. Theoretical analyses have shown that Weber’s law can arise from temporal noise correlations^23^; RNN’s can actively amplify noise through internal feedback likely contributing to Weber’s law.

Weber’s law raises an important question: if independent noise sources cause SD to increase as a function of $\sqrt t$, why does the nervous system settle for Weber’s law^23^? First, it is possible that this reduced accuracy is an unavoidable consequence of the correlated noise^42^. For example, in any neural circuit, slow fluctuations produced by sensory inputs or other brain areas will impose local temporal correlations. Second, the amplification of internal noise may make Weber’s law a necessary cost of the increased computational capacity recurrent neural networks provide.

Why has the Weber-Speed effect not been previously reported? One reason is that most timing studies have relied on interval or duration tasks rather than pattern timing; thus, the Weber coefficient is calculated by fitting the variance of timed responses of distinct intervals collected across blocks. With this approach it is not possible to explicitly examine temporal scaling and the Weber coefficient. In contrast, by studying complex motor patterns consisting of multiple taps^43^, it is possible to estimate the Weber coefficient within each speed, revealing a dependence of the Weber coefficient on speed. As mentioned above this Weber-Speed effect is confounded with the subdivision effect, in which subdividing a target interval into subintervals can improve temporal precision^28,29^. Our results suggest that the subdivision effect may be best reinterpreted as a speed effect. First, in the RNN model the improvement in precision is clearly an effect of speed (of the neural trajectory) because, as implemented here, timing is independent of the behavior of the output unit (e.g., the number of taps). Second, the subdivision hypothesis predicts a sublinear relationship between SD and time, yet a goodness-of-fit analysis revealed that the linear version of Weber’s generalized law generated better fits (Fig. 6 and Supplementary Fig. 9). We thus hypothesize that subdivision effects may in part reflect the speed of the underlying neural trajectories. Specifically, the peak times of rapidly changing signals are less sensitive to independent noise than slower signals (Supplementary Fig. 7)^44^. Future experimental studies, however, will have to further examine the relationship between the Weber-Speed and subdivision effects, and whether the Weber-Speed effect represents a smooth linear transition or discrete steps reflecting different timing mechanisms.

As with Weber’s law, the Weber-Speed effect raises the question of why the nervous system would utilize a timing mechanism that is inherently better—more precise across trials—when engaged in a fast vs. a slow motor pattern. Again, the answer may lie in part in the properties of recurrent circuits. Our analysis of temporal noise correlations revealed larger and longer lasting noise covariance in the RRN during slower trajectories (Supplementary Fig. 10). Additionally, the rate-of-change of a dynamical system and the effects of noise are inversely related^44^. Consider a sinusoidal function at a fast (short period) and slow (long period) speed in the presence of additive noise. If we were to count each peak of the wave’s amplitude as a tic of a clock, additive noise will produce more temporal variance in the peaks of the slow curve because noise added to a slowly changing function is more likely to change the times of the peaks (Supplemental Fig. 7).

The model of temporal scaling presented here makes a number of experimental predictions. The most important prediction, that movements executed at higher speeds are more temporally precise in absolute time, has been tested and confirmed. However, a number of important questions remain, including whether simple interval production tasks correspond to executing the same neural trajectories at different speeds. Two studies indeed suggest that different intervals are timed by similar neural patterns unfolding at different speeds^4,45^. However, no electrophysiological studies have examined temporal scaling during the production of aperiodic temporal patterns similar to those studied here. Additionally, future studies will have to determine if the improved timing with speed observed here is best explained by the actual speed of the underlying dynamics or a subdivision effect.

The model makes a number of additional neurophysiological predictions. First, electrophysiological recordings during temporal scaling to untrained speeds should produce neural trajectories whose positions on a manifold in high-dimensional space reflect the speed of the motor pattern. Second, slower trajectories should exhibit larger temporal noise covariance. In other words, on a trial-by-trial basis, when the population clock reads early at the beginning of a trajectory that deviation will persist longer if the trajectory is moving slowly.

While we propose that the model presented here captures general principles of how neural dynamics account for timing and temporal scaling, the learning rule used to generate the neural trajectories driving timing is not biologically plausible. Future research will have to determine whether such regimes can emerge in a self-organizing manner. However, because the Weber-Speed effect was observed across learning rules, we expect it to be a general property of timing with population clocks (Supplementary Figs. 5, 6). Additionally, while the model is agnostic to what parts of the brain generate such patterns, we hypothesize that similar regimes exist in neocortical circuits characterized by recurrent excitation.

Overall the current studies support the notion that many neural computations can be implemented not by converging to a point attractor^46,47^, but as the voyage through neural phase space^48–50^. And, more specifically, that these trajectories represent dynamic attractors that can encode motor movements and are robust to perturbation—that is, they can return to the trajectory after being bumped off^8^. Here, we show that recurrent neural networks can exhibit regimes with parallel families of neural trajectories that are similar enough to drive the same motor pattern while being traversed at different speeds—accounting for temporal scaling. These regimes predict that the temporal precision of motor responses in absolute time is dependent on speed of execution. This prediction was confirmed in human timing experiments, establishing a novel psychophysical Weber-Speed effect.

## Methods

### Temporal scaling of motor patterns in humans

Human psychophysics experiments were performed using a temporal pattern reproduction task^43^. During the experiments, the subjects sat in front of a computer monitor with a keyboard in a quiet room. On each trial, subjects heard a temporal pattern and then reproduced this pattern by pressing one key on a Cedrus Response Pad™. The target stimulus consisted of a series of brief tones (800 Hz). After the subjects reproduced the pattern, a visual representation of the target and of the subject’s response appeared on the screen along with a score based on the correlation between the target and the reproduced pattern. Stimulus presentation and response acquisition were controlled by a personal computer using custom MATLAB code and the Psychophysics Toolbox^51^. All experiments were run in accordance with the University of California Human Subjects Guidelines.

To test whether temporal scaling is an innate property of motor behavior, subjects we trained to produce the Morse code spelling of “time” at 10 words per minute (Fig. 1). Training occurred over 4 days, with 15 blocks of 15 trials per day. On the fifth day, subjects were asked to produce the trained pattern at 0.5×, 1.0×, and 2× the speed under freeform conditions: subjects first completed 15 trials of the trained pattern, and then were asked to produce the same pattern at the same speed (1×), twice as fast (2×), and twice as slow (0.5×) in the absence of any additionally auditory stimuli. Subjects performed five blocks with five trials per speed in a random order for a total of fifteen trials per block. The subjects were 10 undergraduate students from the UCLA community who were between the ages of 18 and 21. Subjects were paid for their participation.

To test the Weber-Speed prediction of the RNN model (Fig. 5), subjects performed a temporal reproduction task, wherein they heard a pattern of six tones (each lasting 25 ms) and were asked to reproduce the timing of the onset of each tone with a self-initiated start time (representing the first tone). For the 1× speed the six tones were presented at 0, 325, 1025, 1500, 2400, and 3500 ms. This pattern was then scaled to five logarithmically distributed speeds: 0.5×, 0.6×, 1×, 1.5×, and 2.0×. Subjects completed four blocks of fifteen trials per speed in a random order. A pseudo-randomly chosen subset of the subjects were trained to produce the 0.5× and 2× speeds over eight additional days, consisting of ten blocks of fifteen trials per speed. The subjects for this study were 25 undergraduate students from the UCLA community between the ages of 18 and 21 and paid for their participation.

In the periodic/subdivision task (Fig. 6**)** subjects (*n* = 11) were trained on a pattern reproduction tasks in which the four targets consisted of patterns lasting 2.4 seconds divided into subintervals of 300, 400, 600, or 800 ms. Subjects were trained for 5 days and performed four blocks of twelve trials on each condition per day. For the aperiodic timing task in Supplementary Fig. 8, subjects (*n* = 15) reproduced a pattern of six tones presented at 0, 500, 1600, 1950, 2900, and 3500 ms. This pattern was then scaled to speeds 0.5× and 2.0×. Subjects were trained for three days with six blocks of fifteen trials per speed presented in a random order.

### RNN network equations

The units of the RNNs used here were based on a standard firing rate model defined by the equations^21,26^:

1 $$\tau \frac{{{\mathrm{d}}x_i}}{{{\mathrm{d}}t}} = - x_i(t) + \mathop {\sum }\limits_{j \, = \, 1}^N W_{ij}^{{\mathrm{Rec}}}r_j(t) + \mathop {\sum }\limits_{j \, = \, 1}^I W_{ij}^{\mathrm{In}}y_j(t) + \varphi _i(t)$$

2 $$z = \mathop {\sum }\limits_{j \, = \, 1}^N W_j^{{\mathrm{Out}}}r_j$$

where *r*_*i*_ = tanh(*x*_*i*_) represents the output, or firing rate, of recurrent unit *i* = [1,…,*n*]. The variable *y* represents the activity level of the input units, and *z* is the output. *N* = 1800 is the number of units in the recurrent network, and *τ* = 50 *ms* is the unit time constant. The connectivity of the recurrent network was determined by the sparse *NxN* matrix *W*^Rec^, which initially had nonzero weights drawn from a normal distribution with zero mean and SD $g/\sqrt {Np_{\mathrm{c}}}$. The variable *p*_c_ = 0.2 determined the probability of connections between units in the recurrent network, which were drawn uniformly at random, and *g* = 1.6 represents the gain of the recurrent network^21,52^. The *NxI* input weight matrix *W*^In^ was drawn from a normal distribution with zero mean and unit variance. For all figures, *I* = 2, except Supplementary Fig. 2, where additional input units were added to test the specificity of the network response to untrained cue inputs. One input served as cue to start a trial and its activity was set to zero except during the time window −250 ≤ *t* ≤ 0, when its activity was equal to 5.0. The second input unit served as a speed input and was set to a constant level during the time window −250 ≤ *t* ≤ *T*, where *T* represents the duration of the trial. Each unit in the recurrent network was injected with noise current *φ*_*i*_(*t*), drawn independently from a normal distribution with zero mean and SD 0.05, except for the Weber experiments where the SD was 0.25. The recurrent units were connected to the output unit *z* through the *Nx*1 vector *W*^Out^, which was initially drawn from a normal distribution with zero mean and SD $1/\sqrt N$.

### Recurrent learning rule

The networks in this study were trained using the Innate Learning Rule, which trains an initially chaotic recurrent network to autonomously yet reliably produce an arbitrary activity pattern in the presence of noise^8^. It is based on the recursive least squares (RLS) update rule^27,53^. The recurrent weights onto unit *i* were updated every ${\mathrm{\Delta }}t = 5\,{\mathrm{ms}}$ as dictated by

3 $$W_{ij}^{{\mathrm{Rec}}}\left( t \right) = W_{ij}^{{\mathrm{Rec}}}\left( {t - {\mathrm{\Delta }}t} \right) - e_i(t)\mathop {\sum }\limits_{k \in {\boldsymbol{B}}(i)} P_{jk}^i(t)r_k(t)$$

where ***B***(*i*) is the subset of recurrent units presynaptic to unit *i*. The error *e*_*i*_ of unit *i* is given by

4 $$e_i\left( t \right) = r_i\left( t \right) - R_i\left( t \right)$$

where *r*_*i*_ is the firing rate of unit *i* before the weight update, and *R* is the target activity of that recurrent unit. The square matrix ***P***^i^ estimates the inverse correlation of the recurrent inputs onto unit *i*, updated by

5 $${\boldsymbol{P}}^i\left( t \right) = {\boldsymbol{P}}^i\left( {t - {\mathrm{\Delta }}t} \right) - \frac{{{\boldsymbol{P}}^i\left( {t - {\mathrm{\Delta }}t} \right)r\left( t \right)r\prime (t){\boldsymbol{P}}^i\left( {t - {\mathrm{\Delta }}t} \right)}}{{1 + r\prime (t){\boldsymbol{P}}^i\left( {t - {\mathrm{\Delta }}t} \right)r(t)}}$$

### Training procedure

To train a network to perform the temporal scaling task, we first generated a target pattern of recurrent activity by stimulating the network with the cue input and capturing the dynamics generated according to Eq. (1) over 2000 ms in the presence of speed input level *y*_SI_ = 0.3 and zero noise (similar results are obtained if the target pattern is harvested in the presence of noise). We then produced a temporally dilated version of this target by linearly interpolating by a factor of four to produce a second scaled version of the target with a duration of 8000 ms. For Fig. 3 and later, the recurrent network was then trained with random initial conditions and noise amplitude 0.05 according to the algorithm described in Eqs. (3–5). The fast target (2× speed) was trained over the window *t*∈[0,2000] with *y*_SI_ = 0.3 and the slow target (0.5× speed) over the window *t*∈[0,8000] with *y*_SI_ = 0.075. Ten differently seeded networks were each trained for a total of 60 trials alternating between fast and slow targets. A similar procedure was used to train networks at a single speed (Fig. 2). The initial target was captured with a duration of 4000 ms and *y*_SI_ = 0.15 and zero noise. The same initial networks used in the temporal scaling task were trained at this speed for 30 trials. To emulate a rest state all networks were trained to maintain zero *r* (firing rate) for 30 s following the end of each trained recurrent target. We dubbed networks trained in this manner gated attractor networks because they only entered the long-lasting dynamic attractor in response to a specific cued input (Supplementary Fig. 2).

After recurrent training was complete, the output unit was trained, only at the fastest trained speed, to produce a target function of a series of 5 Gaussian peaks (taps) centered at 163, 513, 750, 1200, and 1750 ms (0.5× speed). The training algorithm for the output weights was similar to that used for recurrent training, as described above.

### Analysis of temporal scaling

To assess the ability of a network to generalize its activity to novel speeds, i.e. temporally scale, we tested the response of networks to a range of speed input levels after training was completed (weights were no longer modified). The network was set to a random initial state at *t* = −750 and given the trained cue input during *t*∈[−250,0]. The test speed input was delivered starting at *t* = −250 for a duration lasting 20% longer than when a perfectly timed last tap would occur. The timing of these peaks was used to measure the accuracy of the network’s temporal scaling using a speed factor and scaling index. The speed factor was a coarse measure of temporal scaling calculated by dividing the final peak time of twenty test trials at each speed to the mean peak time at the 1× speed, and taking the mean across trials. The quality of temporal scaling (the scaling index) was calculated by taking the fisher-transformed correlation of the mean timing of the response time for each speed with the mean pattern of the 1× speed.

### Weber analysis

The Weber analysis was performed according to Weber’s Generalized Law^36,54^, which defines a relationship between mean and variance of perceived time as:

6 $$\sigma ^2 = kT^2 + \sigma _{{\mathrm{independent}}}^2$$

where *σ*^2^ is the variance and *T* represent the mean of a given tap time. We define the slope *k* as the Weber coefficient, and $\sigma _{{\mathrm{independent}}}^2$ is the time independent source of variance—sometimes referred to as motor variance. We measured *k* independently at each speed, by performing a linear fit on the measured mean and variance of the five response times at that speed (peak times for the RNN in Fig. 4 and button presses for psychophysics experiments). Note that for visualization purposes, in some plots we show the linear fit of the standard deviation by the mean time.

To test the subdivision hypothesis (Fig. 6), we additionally fit each subject’s responses according to:

7 $$\sigma _{{\mathrm{Subd}}}^2\left( T \right) = k\sum t_i^2 + \sigma _{{\mathrm{independent}}}^2$$

where *t*_*i*_ is the average interval between response *i* and the preceding response. It is important to note that this fit approach was very liberal, because the stronger prediction of the subdivision hypothesis is that it would be possible to fit all the speeds of a subject with a single Weber coefficient—whereas we used different Weber coefficients for each speed (when a single Weber coefficient was used for all speeds the fits were much worse and often did not converge). We then calculated the goodness of fit for both the subdivision and continuous (speed-effect) fits by finding the Fisher-transformed coefficient of determination (*r*^2^) between the predicted variance at each tap time and the measured variance.

### RNN trajectory analysis

To analyze the position of the trajectories in relationship to one another, we tested the networks at each speed without noise. We then concatenated the active period of the trajectory at each speed, defined as the window between cue input offset and speed input offset, and performed PCA on these concatenated trajectories. We used the PCA coefficients to transform the individual trajectory at each speed for visualization in Fig. 7a. To measure the relationship between trajectories, we returned to full (*N* = 1800) neural phase space and measured the Euclidean distance between the slowest (0.5× speed) trajectory and the trajectories at each speed, at all pairs of points in time. This produced one *t*^test^ × *t*^0.5*x*^ distance matrix per speed, as seen in Fig. 7b for test speed 2×. To confirm that the trajectories did not cross and followed a similar path, for each point on the slowest trajectory we found a corresponding point on the test trajectory that was closest to it. This produced a vector of approximately 8000 distance values (for each millisecond of the slowest trajectory) which we plotted in Fig. 7c for each of the five tested speeds. The distances were fairly constant for each test speed and never reached zero, indicating that the trajectories did not intersect. We also recorded the points $t_{{\mathrm{min}}}^{{\mathrm{test}}}$ along the test trajectory where these minima occurred, allowing us to assess the relative speed of each trajectory along their entire length. For example, when the slowest trajectory is at its 400 ms mark, if a test trajectory is closest to it at the test trajectory’s own 100 ms mark, this would indicate that at that moment, the slowest trajectory was moving four times slower than the test trajectory. We plotted $t_{{\mathrm{min}}}^{{\mathrm{test}}}$ for each of the five tested speeds in Fig. 7d.

### Recurrent-decay-input subspace decomposition

In Fig. 8, the total drive$( {\frac{{{{\mathrm{d}x}}({{t}})}}{{{{\mathrm{d}t}}}},{\mathrm{Equation}}\,1} )$ was decomposed into its three components: (1) synaptic decay$\left( {{\mathbf{DS}}(t) = - \frac{1}{\tau }{\boldsymbol{x}}(t)} \right)$; (2) recurrent synaptic drive$\left( {{\mathbf{RS}}(t) = \frac{1}{\tau }{\mathbf{W}}^{\mathrm{Rec}}{\boldsymbol{r}}(t)} \right)$; and its external component, the tonic speed input$\left( {{\mathbf{IS}}(t) = \frac{1}{\tau }{\mathbf{W}}^{\mathrm{In}}{\boldsymbol{y}}(t)} \right)$. The magnitude of each of these components was calculated as the time-averaged L2-norm of the corresponding population vectors. Figure 8c illustrates the generation of an orthonormal basis set {***is***, ***ds***, ***rs***} for the total drive at time *t*, which was computed by applying the Gram-Schmidt orthonormalization process as follows:

8 $${\mathbf{is}} = \frac{{{\mathbf{IS}}(t)}}{{\left\| {{\mathbf{IS}}(t)} \right\|}}$$

9 $${\mathbf{ds}} = \frac{{{\mathbf{DS}}\left( {{t}} \right) - \left( {{\mathbf{DS}}\left( {{t}} \right)\prime {\mathbf{is}}} \right){\mathbf{is}}}}{{\left\| {{\mathbf{DS}}\left( {{t}} \right) - \left( {{\mathbf{DS}}\left( {{t}} \right)\prime {\mathbf{is}}} \right){\mathbf{is}}} \right\|}}$$

10 $${\mathbf{rs}} = \frac{{{\mathbf{RS}}\left( {{t}} \right) - \left( {{\mathbf{RS}}\left( t \right)\prime {\mathbf{is}}} \right){\mathbf{is}} - \left( {{\mathbf{RS}}\left( t \right)\prime {\mathbf{ds}}} \right){\mathbf{ds}}}}{\left\|{{{\mathbf{RS}}\left( {{t}} \right) - \left( {{\mathbf{RS}}\left( t \right)\prime {\mathbf{is}}} \right){\mathbf{is}} - \left( {{\mathbf{RS}}\left( t \right)\prime {\mathbf{ds}}} \right){\mathbf{ds}}}}\right\|}$$

Here, ||.|| represents the L2-norm and the apostrophe represents the vector transpose operation. Collectively, these unit orthonormal vectors fully describe the total drive and its components at *t*, and therefore, form a basis set for these vectors. The plane described by the basis set {***ds***, ***rs***} is denoted the internal drive plane, with ***DS***(*t*) projected onto this plane in gray, and ***RS***(*t*) in yellow. In Fig. 8d, we visualize the relationship between these vector projections over a short sequence of time steps along the slow and fast trajectories, on a common internal drive plane. For this, we constructed a common orthonormal set by applying the Gram-Schmidt process to the sequence-averaged component vectors. While doing so precludes the orthonormal set from forming a basis for the vector sequences, restricting the length of these sequences to a small fraction of the network unit time constant (*τ*), renders the information loss negligible. Finally, in Fig. 8e, to show that the trajectories consistently encode their desired speeds, we plot the projection of the state variable (***x***(*t*)) onto ***is***, against its projection onto the first principal component in the subspace orthogonal to ***is***. That is, the *x*-axis represents the first principal component of (***x***(*t*) − (***x***(*t*)′***is***)***is***).

### Temporal noise analysis

In Supplementary Figs. 4 and 10, we evaluated temporal noise statistics of the RNN trajectories to determine the basis of their adherence to Weber’s law. The temporal noise within a trajectory during trial *k*, ***r***^***k***^, was calculated relative to the trial-averaged trajectory at the corresponding speed, $\overline {\boldsymbol{r}}$. Specifically, the temporal noise within ***r***^***k***^ relative to $\overline {\boldsymbol{r}} (t)$ was calculated as *η*^*k*^(*t*) = *t* − *t*′ where ***r***^***k***^*(t*′*)* was the point along ***r***^***k***^ closest to $\overline {\boldsymbol{r}} (t)$. Since measurements showed that the temporal noise within the trajectories exhibited a time-varying standard deviation (i.e. it was non-stationary, Supplementary Fig. 4b), the auto-correlation between the temporal noise at time points *s* and *t* was calculated as:

11 $$\frac{{\frac{1}{K}\mathop {\sum }\nolimits_{k = 1}^K \left( {\eta ^k\left( t \right) - \mu _\eta \left( t \right)} \right)\left( {\eta ^k\left( s \right) - \mu _\eta \left( s \right)} \right)}}{{\sigma _\eta (t)\sigma _\eta (s)}}$$

where *μ*_*η*_(*t*) and *σ*_*η*_(*t*) symbolize the sample mean and standard deviation of the temporal noise at time *t*. However, since the mean temporal noise did not vary with time, the auto-covariance at lag *τ* was calculated as:

12 $$\mathop {\sum }\limits_{k = 1}^K \left( {\eta ^k\left( t \right) - \mu _\eta } \right)\left( {\eta ^k\left( {t + \tau } \right) - \mu _\eta } \right)$$

### Control networks

We trained five control RNNs using Hessian-free optimization^24,25^ to produce the same aperiodic output pattern as RNNs trained using the innate learning rule, at the 0.5× and 2× speeds. These networks were defined by:

13 $$\tau \frac{{{\mathrm{d}}x_i}}{{{\mathrm{d}}t}} = - \,x_i(t) + \mathop {\sum }\limits_{j = 1}^N W_{ij}^{{\mathrm{Rec}}}r_j(t) + \mathop {\sum }\limits_{j = 1}^I W_{ij}^{\mathrm{In}}y_j\left( t \right) + b_i^x + \varphi _i\left( t \right)$$

14 $$z = \mathop {\sum }\limits_{j = 1}^N W_{ij}^{{\mathrm{Out}}}r_j + b^z$$

where network size is *N* = 300 and *r*_*i*_ = tanh(*x*_*i*_) is the firing rate of recurrent unit *i* = [1,…,*N*]. As in the innate learning RNNs, there was a cue and speed input, and Gaussian noise *φ*_*i*_(*t*) drawn from a normal distribution with SD 0.25. The Hessian-free learning algorithm adjusts the recurrent weights *W*^Rec^ by backpropagating the error in the output unit during a trial across *W*^Rec^, defined as *e*_*i*_(*t*) = *z*(*t*) − *Z*(*t*), where *Z* is target output activity. Training resulted in the modification of bias terms *b*^*x*^ and *b*^*z*^, and the weight matrices *W*^*In*^, *W*^Rec^, and *W*^Out^. In this study, *W*^Rec^ was fully connected, unlike the sparsely connected RNNs used elsewhere. Networks trained for the simplified output target in Supplementary Fig. 6e, f had network size *N* = 100. Other parameters were the same as in the innate learning studies. The code used to train these networks was based on Dr. David Sussillo’s Hessian-free optimization implementation in MATLAB available at: https://github.com/sussillo/hfopt-matlab.

We also trained three Echo State Networks^26,27^ (ESNs) to produce a sinusoidal outputs (ESN’s are not well-suited to produce long aperiodic patterns) at three different frequencies: 5, 10, and 15 Hz. ESNs have a similar architecture, except there is feedback from the output unit to the recurrent units. These networks were governed by the equations:

15 $$\tau \frac{{{\mathrm{d}}x_i}}{{{\mathrm{d}}t}} = - x_i(t) + \mathop {\sum }\limits_{j = 1}^N W_{ij}^{{\mathrm{Rec}}}r_j(t) + W_i^{\mathrm{In}}y\left( t \right) + W_i^{{\mathrm{FB}}}z(t) + \varphi _i(t)$$

16 $$z = \mathop {\sum }\limits_{j = 1}^N W_{ij}^{{\mathrm{Out}}}r_j$$

which are the same as those of the innate learning RNNs, but with the additional feedback term $W_i^{\mathrm{FB}}$ defining the weight of the feedback from output *z* onto recurrent unit *x*_*i*_. The networks size was set to *N* = 300, and *W*^FB^ was drawn from a uniform distribution on the open interval (−1, 1) and delivered feedback to each unit in the recurrent network. As before, *W*^In^ and *W*^Out^ were drawn from a normal distribution with zero mean and unit variance for *W*^In^ and SD $1/\sqrt N$ for *W*^Out^. Recurrent weights *W*^Rec^ were drawn from a normal distribution with zero mean and SD $g/\sqrt {Np_{\mathrm{c}}}$, with gain *g* = 1.2 and connection probability *p*_c_ = 0.2. These networks were trained by modifying the weights onto the outputs units to match the output target *Z* based on the error t*e*rm *e*_*i*_(*t*) = *z*(*t*) − *Z*(*t*)(using the FORCE algorithm^27^). During training and testing, the networks received a single input *I* of amplitudes 1.2, 1, or 0.8 which determined the target output frequency, with higher amplitudes corresponding to higher frequency.

## Electronic supplementary material

Supplementary Information

## Data Availability

Data and code used to generate the main simulation in this manuscript will be made available upon request, or code can be downloaded from: https://github.com/nhardy01/RNN.
