# Supplementary material for: A model of temporal scaling correctly predicts that motor timing improves with speed
Source: Nat Commun. 2018 Nov 9;9:4732. doi: 10.1038/s41467-018-07161-6 (PMC6226482; doi:10.1038/s41467-018-07161-6)
Supplement: Supplementary file 1 — Supplementary Information [file 41467_2018_7161_MOESM1_ESM.pdf]

NCOMMS-18-15378A

A Model of Temporal Scaling Correctly Predicts that Motor Timing Improves with Speed

Hardy et al

Supplementary Information

SUPPLEMENTARY FIGURES

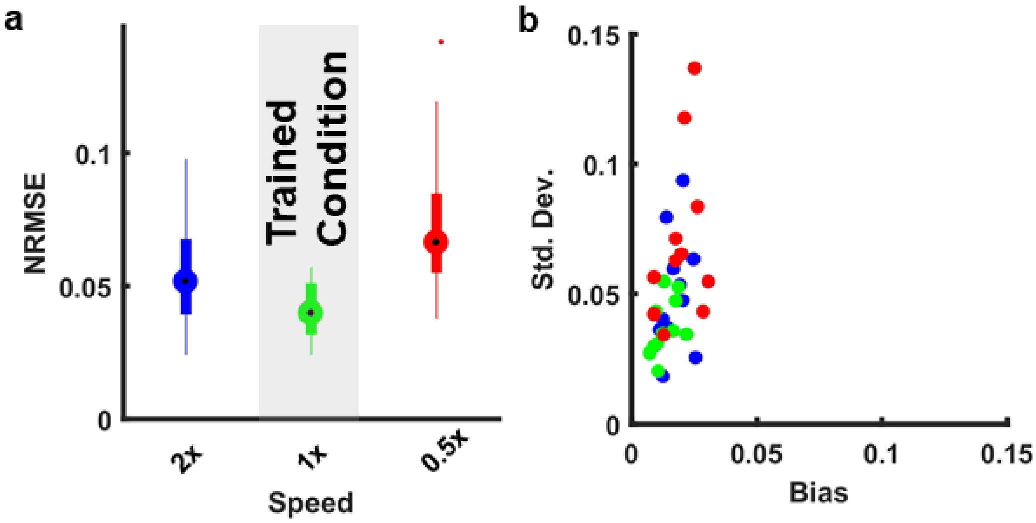

Supplementary Figure 1. Performance on the Morse code task based on normalized RMSE. **a)** Normalized RMSE was calculated in relation to the target pattern and based on the normalized response times (normalized to the mean response time of the last response at each condition). As shown in **Fig. 1** subjects produced a range of different speeds—thus the actual speeds within a given group can vary significantly. There was a trend toward a significant difference between NRMSE between 2x and the trained 1x groups (t_{10}=2, p=0.073, paired t-test) and a significant difference between 0.5x and 1x (t_{10}=2, p=0.004, paired t-test). Note that the 2x and 0.5x labels reflect requested scaling factors, not actual produced speeds, thus this data must be interpreted with caution. **b)** Scatterplot of the mean SD (across responses) and mean biases (difference between the target response time and mean response time across all responses) for each subject on each condition.

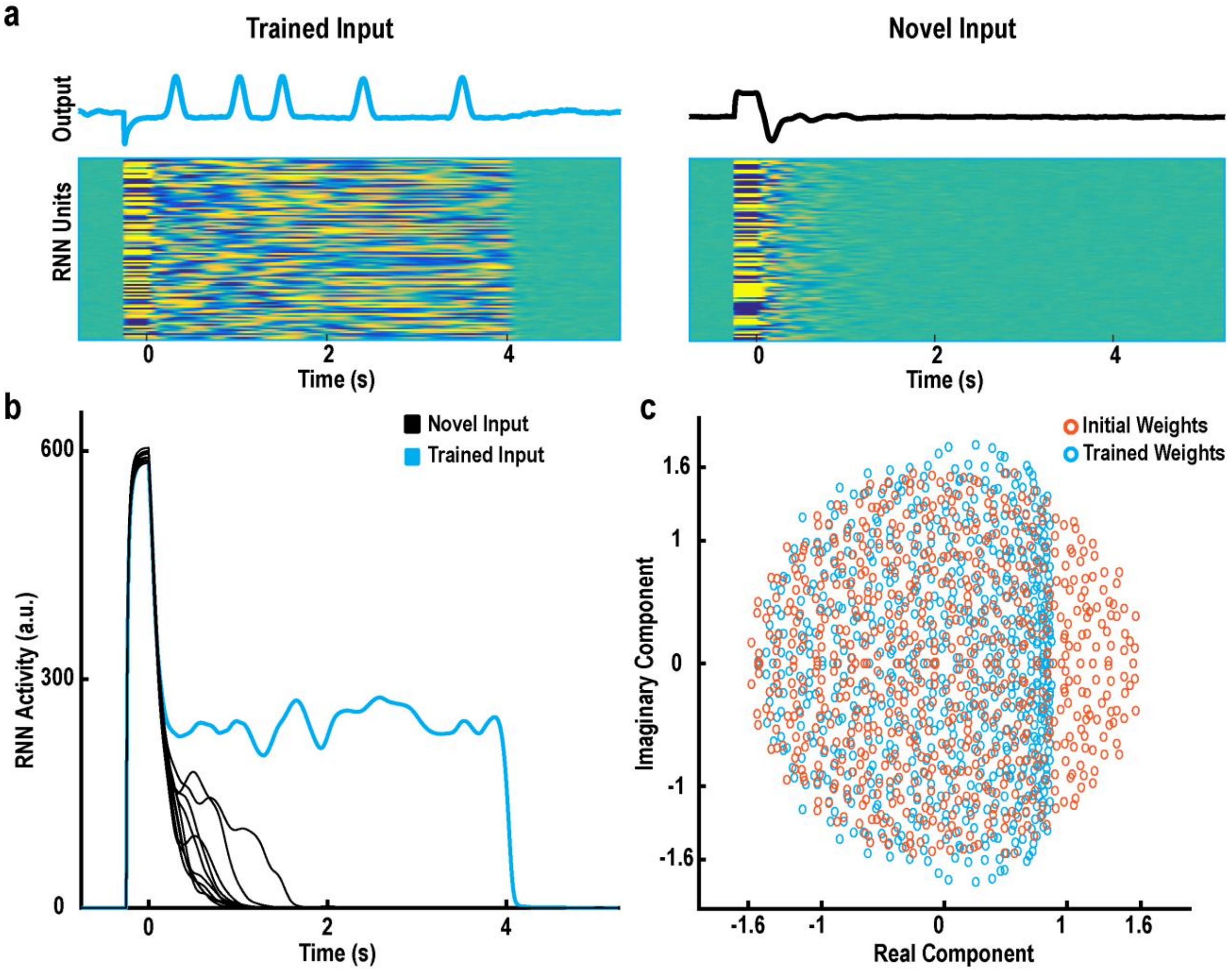

Supplementary Figure 2. Gated attractor networks suppress untrained activity.
a) RNNs were trained to suppress activity except in response to trained cue inputs. Left: Example activity after training in response to the trained cue input. Right: Response to an untrained cue. **b)** RNN activity (the norm of the firing rate (r) in response to the trained cue and ten untrained cue inputs. **c**) The eigenvalues of the recurrent weights before and after training. After training, the real components of the eigenvalues are less than one, meaning gated attractor networks are not spontaneously active.

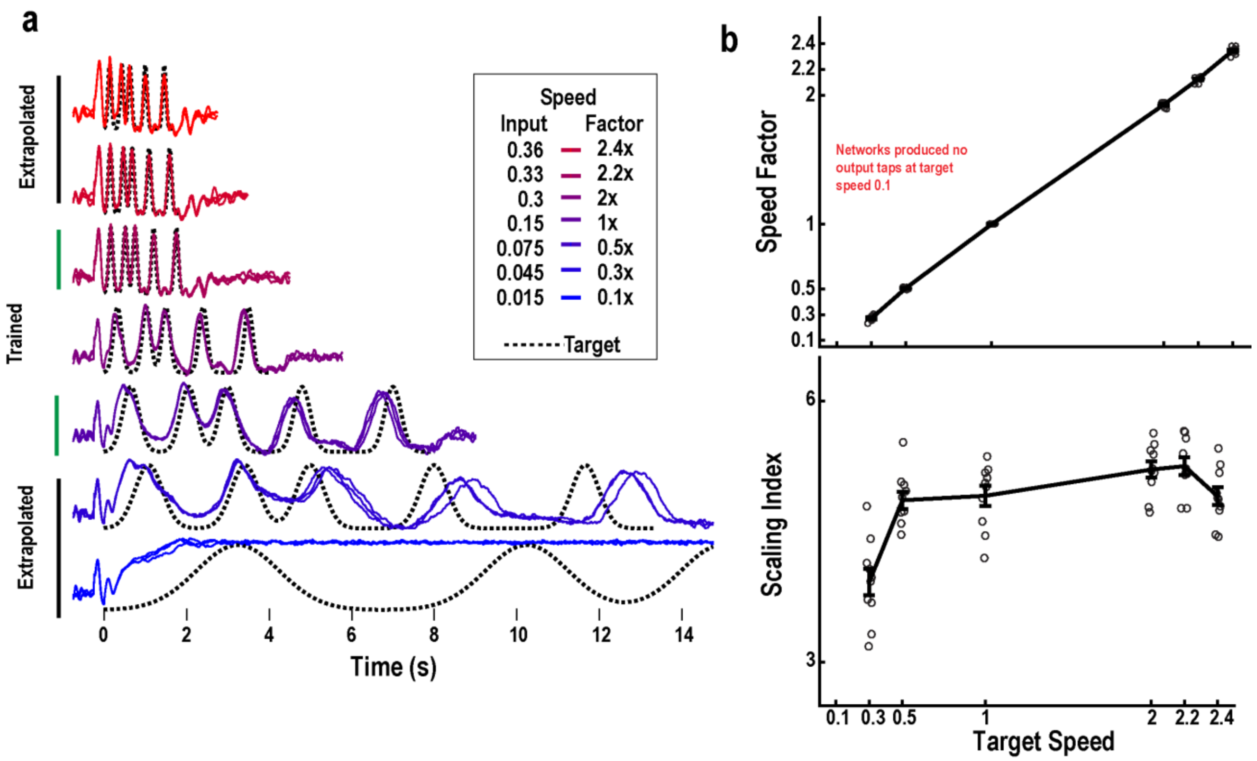

Supplementary Figure 3. RNNs' temporal scaling degrades outside of the trained speed range. **a)** Output traces at interpolated and extrapolated speeds (outside of the trained speed range). **b)** Top: speed factor (top) and scaling index (bottom) of ten networks calculated from twenty trials at the speeds shown in **a**. Generalization degrades at slower speeds. Error bars indicate SEM (n = 10).

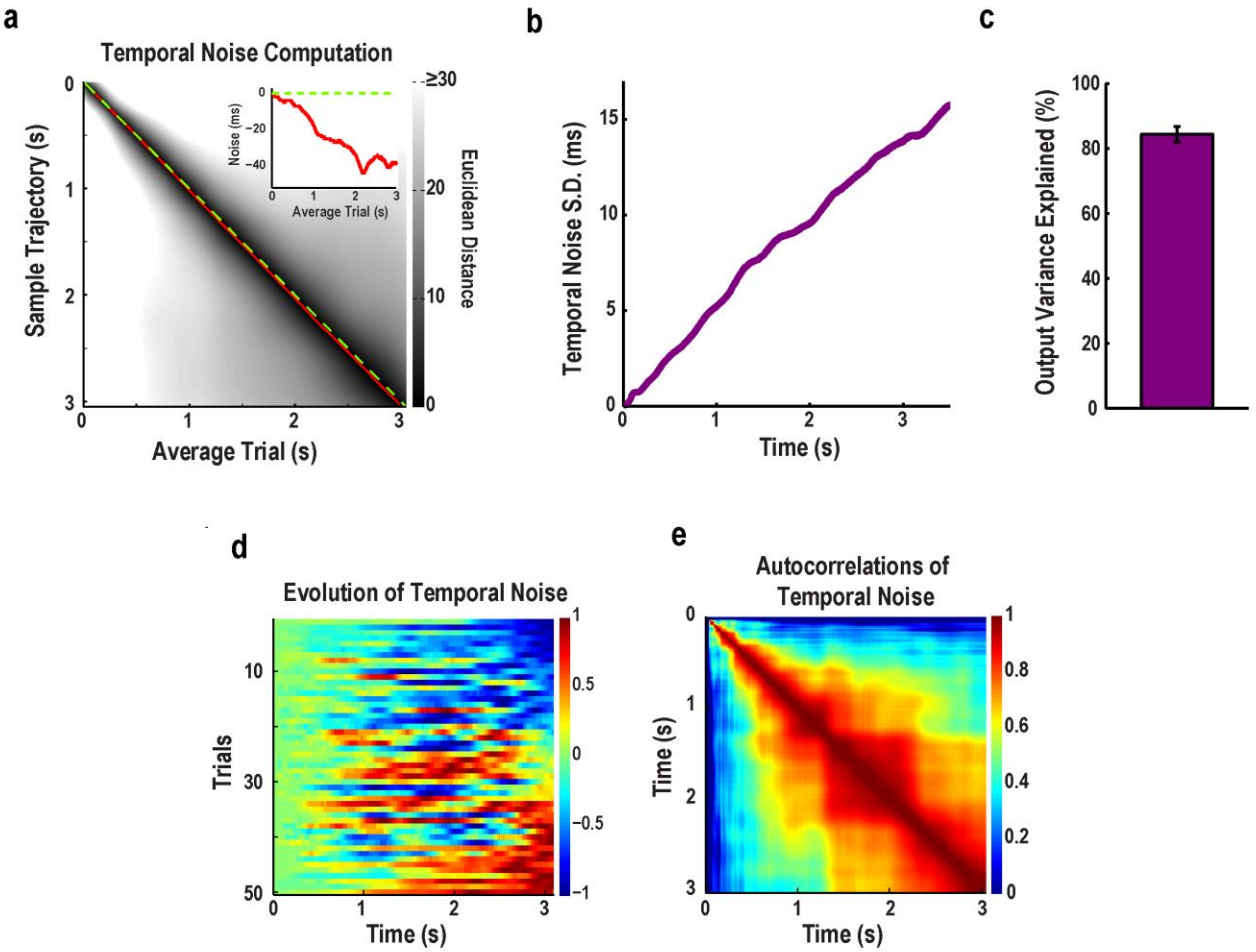

Supplementary Figure 4. RNNs produce long-lasting temporal noise correlations.
a) Euclidean distance matrix between the trial-averaged trajectory of a trained RNN and a single trial at speed 1x. The times at which the two trajectories are closest is represented by the red line. Matched time points between the trajectories is represented by the identity line (green dashed line). Over time the sample trajectory runs further ahead of the average trajectory (temporal noise), as evidence by the red line being below the green line. Inset: Temporal noise (red line) in the sample trajectory relative to the average trajectory. At the end of the average trajectory, the sample trajectory is ~40 ms ahead. **b)** The linear relationship of the SD of temporal noise in the trajectories and absolute time underlies Weber's law observed in the output unit. The SD of temporal noise is calculated over 50 trials, averaged across 10 networks. **c)** Output timing variability explained by temporal noise in the RNN (normalized mean squared error calculated between temporal noise in the trajectories and the output), averaged across 10 networks (error bars indicate SEM). **d)** Normalized temporal noise across 50 trials, sorted according to the noise at the end of the trajectory in the example network. **e)** Autocorrelation of temporal noise in one network. Each element in the matrix represents the correlation (across trials) of temporal noise at the corresponding pair of time points (i.e., pair of columns in **d**). Deviations at early time points predict later deviations. Networks (n=10) trained and tested at noise amplitude of 0.25.

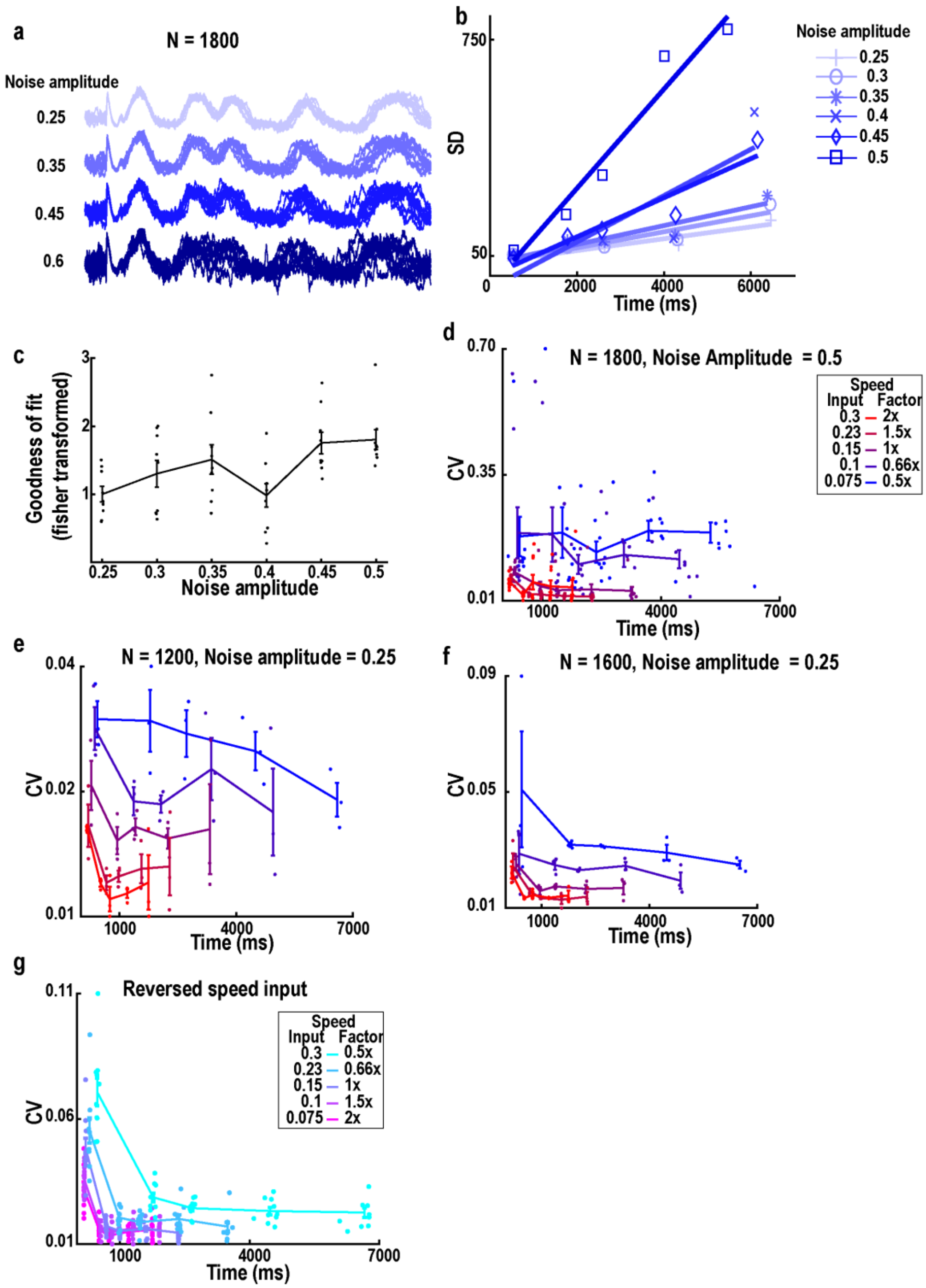

Supplementary Figure 5. Weber-speed effect in RNNs is observed across noise levels and network size.

a) Ten example output traces from a single network at increasing levels of noise. The output pattern is discernable over a range of noise amplitudes (<0.5). **b)** SD vs t of hit times across noise amplitudes for the example network. Solid lines show the linear fits and symbols show the measured statistics. **c)** The Weber's law (linearity of σ^2 vs t) is maintained within this stable range. Beyond a noise amplitude of 0.5 the output becomes too unsteady to reliably measure the hit time. **d-f)** The Weber- speed effect persists in high noise (panel d), and lower network size (n=3) (panels e, f). **g)** The Weber-speed effect is also observed when

RNNs (n = 10) are trained with an inverse speed input amplitude vs. speed relationship. Error bars indicate SEM.

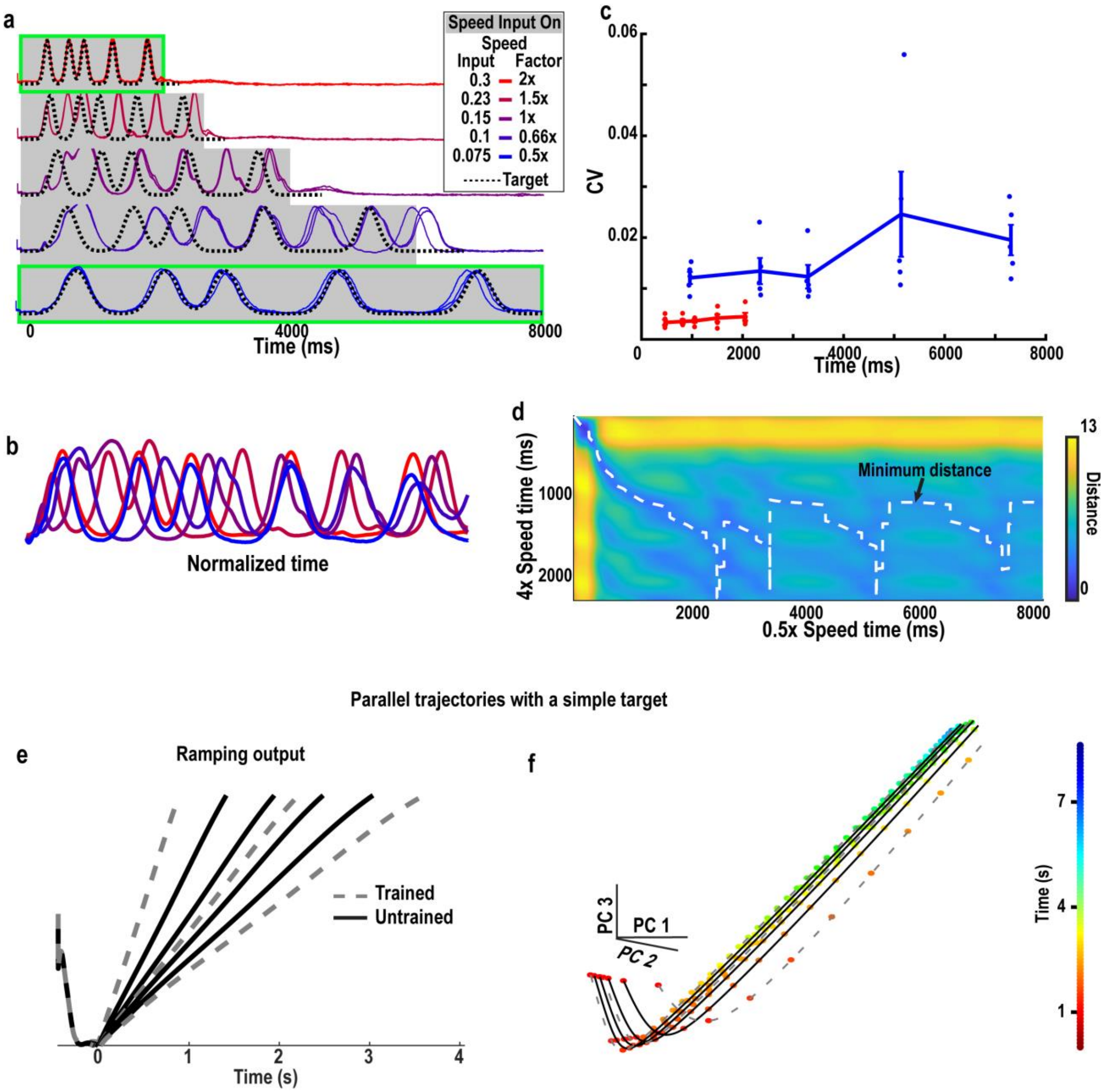

Supplementary Figure 6. RNN training based on output error does not result in robust temporal scaling.
Four control networks were trained at 0.5x and 2x speeds using the Hessian-free backprop algorithm, using the same speed-input relationship as the innate learning studies. **a)** Using the same training parameters used in the main text (i.e., training on only 2 speeds, and with the same noise levels) Hessian-free backprop did not result in temporal scaling to novel speeds (note the difference in the number of peaks). Three traces from an example network are shown at interpolated and trained speeds. **b)** Output at the trained speeds in normalized time. **c)** The Weber-speed effect is still observed at the two trained speeds, despite the absence of generalization to interpolated speeds. **d)** Pairwise Euclidean distance between the network trajectories at the trained speeds. The trajectories follow different paths (i.e. they are not parallel) as shown by the jagged trace of the minimum distance between the two speeds (white dashed line). **e)** Networks trained for a simple ramping output generalize to novel speeds, and **f)** produce parallel trajectories across speeds.

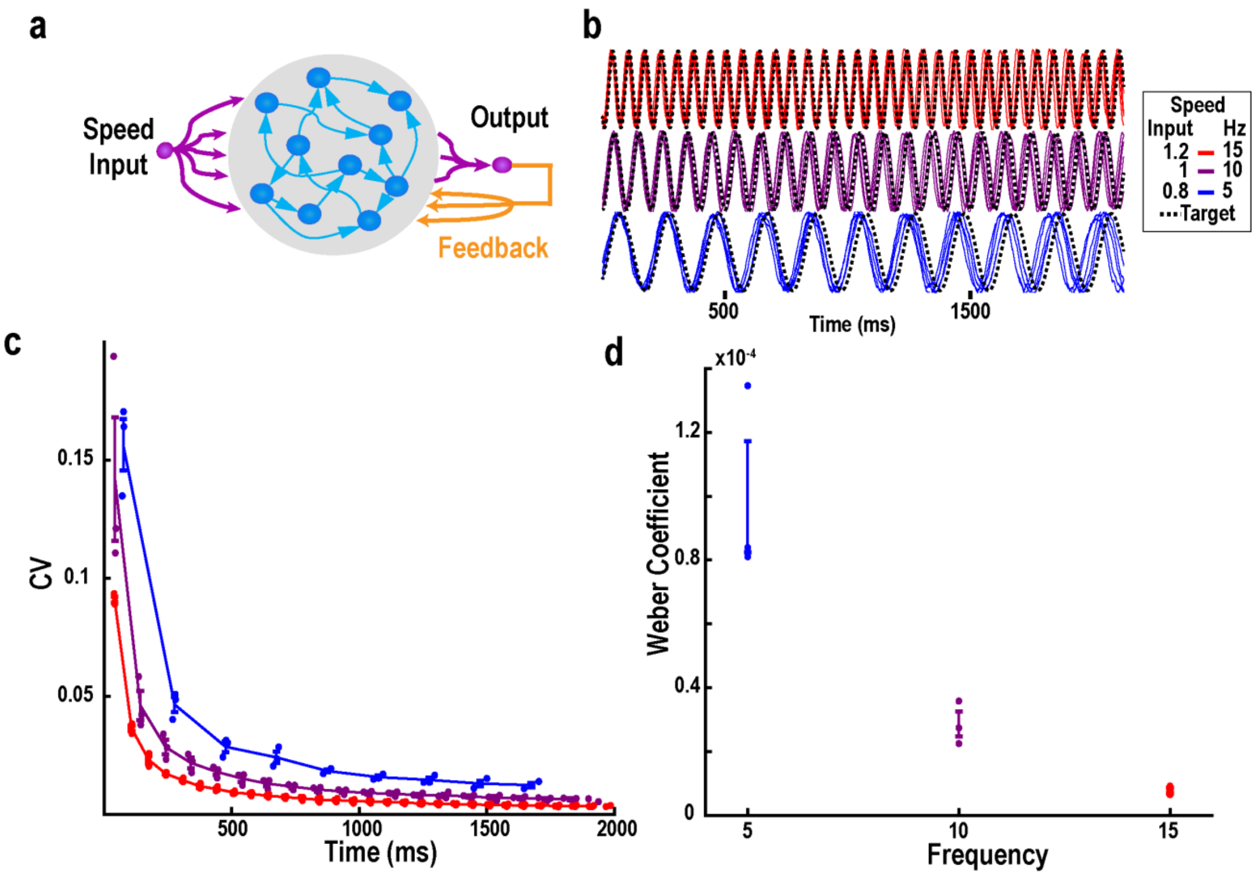

Supplementary Figure 7. Echo state network the produces sinusoids of different frequencies exhibits the Weber-speed effect.

a) Network schematic. The recurrent network receives a speed input, and generates a single output as in the other architectures. However, the output unit now provides feedback onto the recurrent units, and only the weights onto the output unit are trained to produce a sinusoid. **b)** Output targets and 5 example traces for each frequency. Networks were trained to produce sinusoidal output with frequencies 5, 10, and 15 Hz and input amplitudes 0.8, 1, and 1.2 by modifying the recurrent-to-output synapses (Methods). **c)** The coefficient of variation (Weber factor) and **d)** Weber Coefficient demonstrate reduced variability at higher frequencies (the Weber-speed effect). Error bars indicate SEM (n=3)

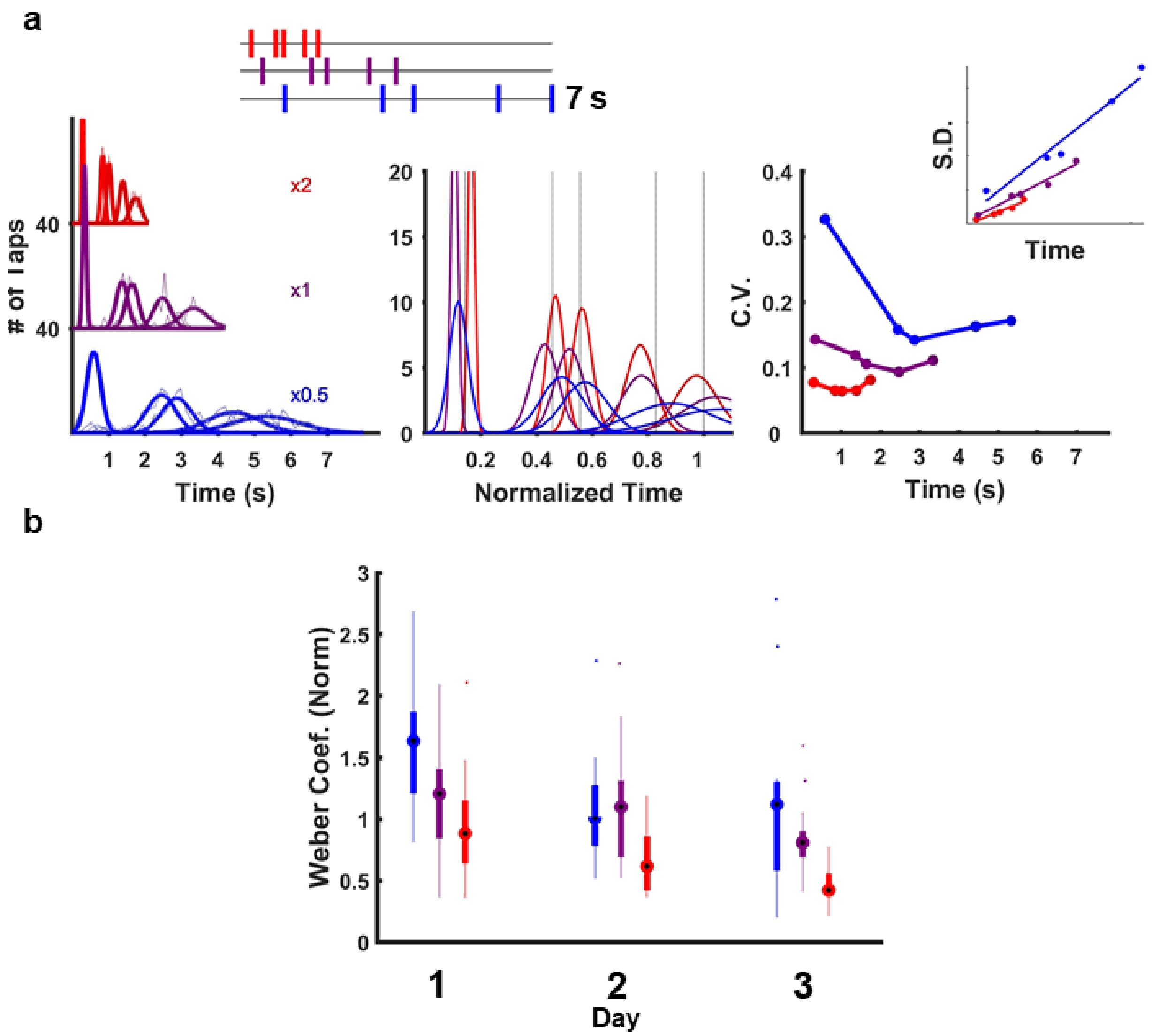

Supplementary Figure 8. Weber-speed effect on an aperiodic task composed of three speeds in which subjects were trained across three days on all speeds.
a) Left: Histogram (dashed lines) and Gaussian fits (solid lines) of the taps at all three speeds (0.5x, 1x, and 2x) from a single subject. Middle: the fits shown with time normalized to the mean of the last tap (vertical lines represent target times). Right: CV of each tap at each speed, with the linear fig of the SD versus mean time plotted in the inset. **b)** Whisker plots of the CV of all subjects (n=15) for all three speeds across the three days of training.

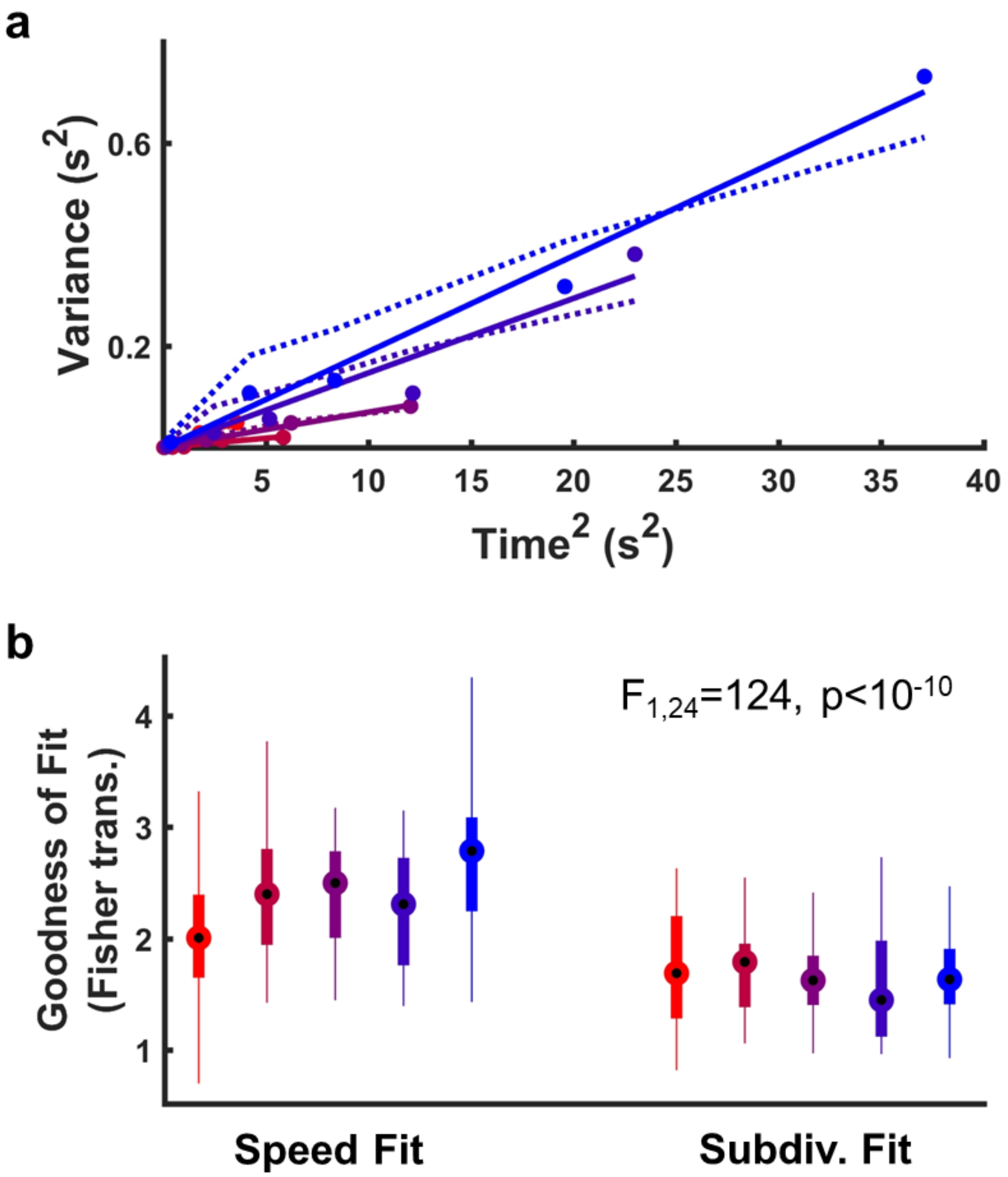

Supplementary Figure 9. Comparison of the Weber-speed and subdivision hypotheses for the aperiodic task.

Analysis based on the data of the 25 subjects presented in Figure 5. **a)** Example fits of the variance at time *T* composed of n subintervals (t_1, $t_{2, \ldots,}$ t_n) according to the speed (continuous, solid lines) and subdivision (reset, dashed lines) hypotheses (σ_{ind}^2 represents the time independent source of variance). **b)** Goodness of fit values (Fisher transformed r^2) for both the speed and subdivision hypotheses for each speed across all subjects (repeated measures ANOVA).

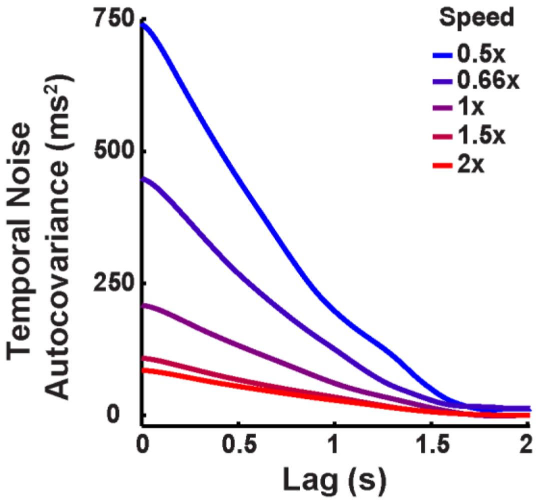

Supplementary Figure 10. Slower speeds have longer lasting noise correlations. Autocovariance of temporal noise at different speeds across 50 trials, averaged across 10 networks; note that the slowest speed has an elevated covariance even at a time lag of up to 1 s. Networks trained and tested at noise amplitude of 0.25.
